# Composition of active bacterial communities and presence of opportunistic pathogens in disinfected and non-disinfected drinking water distribution systems in Finland

**DOI:** 10.1016/j.watres.2023.120858

**Published:** 2023-11-11

**Authors:** Sallamaari Siponen, Balamuralikrishna Jayaprakash, Anna-Maria Hokajärvi, Vicente Gomez-Alvarez, Jenni Inkinen, Ivan Ryzhikov, Pia Räsänen, Jenni Ikonen, Anna Pursiainen, Ari Kauppinen, Mikko Kolehmainen, Jussi Paananen, Eila Torvinen, Ilkka T. Miettinen, Tarja Pitkänen

**Affiliations:** a Finnish Institute for Health and Welfare, Department of Health Security, P.O. Box 95, 70701 Kuopio, Finland; b University of Eastern Finland, Department of Environmental and Biological Sciences, P.O. Box 1627, 70211 Kuopio, Finland; c U.S. Environmental Protection Agency, Office of Research and Development, 26W. Martin Luther King Dr., Cincinnati, OH 45268, United States; d University of Eastern Finland, Institute of Biomedicine, P.O. Box 1627, 70211 Kuopio, Finland; e University of Helsinki, Faculty of Veterinary Medicine, Department of Food Hygiene and Environmental Health, P.O. Box 66, 00014 Helsinki, Finland

**Keywords:** Bacterial communities, Disinfection, Drinking water, Opportunistic pathogens, Ribosomal RNA

## Abstract

Many factors, including microbiome structure and activity in the drinking water distribution system (DWDS), affect the colonization potential of opportunistic pathogens. The present study aims to describe the dynamics of active bacterial communities in DWDS and identify the factors that shape the community structures and activity in the selected DWDSs. Large-volume drinking water and hot water, biofilm, and water meter deposit samples were collected from five DWDSs. Total nucleic acids were extracted, and RNA was further purified and transcribed into its cDNA from a total of 181 water and biofilm samples originating from the DWDS of two surface water supplies (disinfected with UV and chlorine), two artificially recharged groundwater supplies (non-disinfected), and a groundwater supply (disinfected with UV and chlorine). In chlorinated DWDSs, concentrations of <0.02–0.97 mg/l free chlorine were measured. Bacterial communities in the RNA and DNA fractions were analysed using Illumina MiSeq sequencing with primer pair 341F-785R targeted to the 16S rRNA gene. The sequence libraries were analysed using QIIME pipeline, Program R, and MicrobiomeAnalyst. Not all bacterial cells were active based on their 16S rRNA content, and species richness was lower in the RNA fraction (Chao1 mean value 490) than in the DNA fraction (710). Species richness was higher in the two DWDSs distributing non-disinfected artificial groundwater (Chao1 mean values of 990 and 1 000) as compared to the two disinfected DWDSs using surface water (Chao1 mean values 190 and 460) and disinfected DWDS using ground water as source water (170). The difference in community structures between non-disinfected and disinfected water was clear in the beta-diversity analysis. Distance from the waterworks also affected the beta diversity of community structures, especially in disinfected distribution systems. The two most abundant bacteria in the active part of the community (RNA) and total bacterial community (DNA) belonged to the classes *Alphaproteobacteria* (RNA 28 %, DNA 44 %) and *Gammaproteobacteria* (RNA 32 %, DNA 30 %). The third most abundant and active bacteria class was *Vampirovibrionia* (RNA 15 %), whereas in the total community it was *Paceibacteria* (DNA 11 %). Class *Nitrospiria* was more abundant and active in both cold and hot water in DWDS that used chloramine disinfection compared to non-chlorinated or chlorine-using DWDSs. Thirty-eight operational taxonomic units (OTU) of *Legionella*, 30 of *Mycobacterium*, and 10 of *Pseudomonas* were detected among the sequences. The (RT)-qPCR confirmed the presence of opportunistic pathogens in the DWDSs studied as *Legionella* spp. was detected in 85 % (mean value 4.5 × 10^4^ gene copies/100 ml), *Mycobacterium* spp. in 95 % (mean value 8.3 × 10^6^ gene copies/100 ml), and *Pseudomonas* spp. in 78 % (mean value 1.6 × 10^5^ gene copies/100 ml) of the water and biofilm samples. Sampling point inside the system (distance from the waterworks and cold/hot system) affected the active bacterial community composition. Chloramine as a chlorination method resulted in a recognizable community composition, with high abundance of bacteria that benefit from the excess presence of nitrogen. The results presented here confirm that each DWDS is unique and that opportunistic pathogens are present even in conditions when water quality is considered excellent.

## 1. Introduction

Drinking water microbial quality may change after leaving the waterworks and before reaching the user’s tap in a drinking water distribution system (DWDS) (Potgieter et al., 2018; Hayward et al., 2022). Drinking water quality in European Union member states must fulfill microbial and chemical requirements and not contain feacal contamination indicating nor pathogenic microbes (EU, 2020). In the future, also risk assessment on the building’s water system is required in so called priority premises, such as hospitals and hotels, to assess whether the risk for the presence of *Legionella* has increased (EU, 2020). The selection of drinking water treatment techniques and disinfection affects the microbial content of the water going from the waterworks into the distribution system, but microbial nutrients from source water, pipe material, temperature, and the age of the water may also affect the microbiome composition and activity in water and biofilms on the inner surfaces of the pipes in many complex ways (Potgieter et al., 2018; Neu and Hammes, 2020; Leslie et al., 2021). Biofilms also give shelter to bacteria, including opportunistic pathogens, that may not be detected abundant in water when DWDS is well maintained (Waak et al., 2019; Leslie et al., 2021; Rosenqvist et al., 2023).

Disinfection residual is used to maintain good quality (WHO, 2022) and disinfection is always required when surface water is used as source water in Finland (STM, 2015). While most of the published results about bacterial communities in DWDSs concern chlorinated drinking water systems, bacterial communities in non-chlorinated DWDSs are less studied (Potgieter et al., 2018; Bruno et al., 2018; Rosenqvist et al., 2023). It is known that disinfection residual in DWDSs causes selective pressure on microbiota and decreases diversity in bacterial communities and their functional potential compared to non-disinfected water systems (Waak et al., 2019; Dai et al., 2020). Also, changes in water temperatures shifts bacterial composition (Ji et al., 2017; Dai et al., 2018). High hot water temperatures may decrease bacterial diversity compared to cold water (Zhang et al., 2021), but complex bacterial communities in full-scale hot water systems are not yet well understood.

In addition to general changes in microbial numbers and composition, disinfection may also affect the colonization of opportunistic pathogen microbes (Leslie et al., 2021; Hayward et al., 2022) by promoting the occurrence of some bacteria species more than others (Donohue et al., 2019). Several studies show that the use of chloramine may promote the occurrence of *Mycobacterium* species or may be less efficient against *Mycobacterium* spp. compared to free chlorine compounds, whereas *Legionella* spp. have been found to be more common in drinking water exposed to free chlorine compounds than in water disinfected using chloramine (Donohue et al., 2019; Gomez-Alvarez et al., 2012; Wang et al., 2013), although not all drinking water system studies support this finding (Buse et al., 2019; Bautista-de los Santos et al., 2016). Survival of opportunistic pathogens, such as *Legionella*, may be favored also by the temperature prevailing in hot water systems. Temperatures over 50 °C are needed to control growth of many bacteria, including opportunistic pathogens (Proctor et al., 2017; Dai et al., 2020; Leslie et al., 2021; Hayward et al., 2022), but this temperature is not always reached in hot water systems (Schwake et al., 2016).

The presence of viable, metabolically active bacteria belonging to genera capable of causing infection may pose a health risk to water consumers, and should be differentiated from dead cells or free DNA of pathogenic bacteria that are not able to infect humans. Recent studies have used ribosomal RNA as a target of microbial community studies to reveal the active fraction of microbes (Pitkänen et al., 2013; Inkinen et al., 2018, and 2019). An RNA-based approach gives an estimation of live (either dormant or metabolically active) members of the bacterial community, as ribosomal RNA is actively produced and regulated by living cells and it degrades more quickly than DNA after cell death (Li et al., 2017). In DWDSs, living members of bacterial communities and their activity are less studied compared to studies using DNA-based methods (Potgieter et al., 2018; Bruno et al., 2018; Waak et al., 2019; Rosenqvist et al., 2023).

The objective of this study was to characterize active and total bacterial community members and their richness and diversity in cold and hot water, and biofilms of pipes and water meters in five well-maintained full-scale DWDSs based on 16S rRNA and 16S rRNA gene analysis. We aimed to determine the effects of distance in distribution system, season of the year, and water quality parameters on the bacterial communities and their activity. Our objective was also to discover the presence and activity of opportunistic pathogens in cold and hot water and biofilms in the disinfected and non-disinfected DWDSs studied. The overarching goal was to assess the effects of raw water source, different treatment/disinfection methods, and environmental factors on microbiomes in drinking water and biofilms, providing information regarding potential future options to limit the growth potential of harmful microbes within the microbiomes of DWDSs.

## 2. Materials and methods

### 2.1. Water sampling

In this study, molecular methods were used to analyze the dynamics of bacterial communities of five DWDSs in Finland. The waterworks studied, described earlier by Ikonen et al. (2017) and Inkinen et al. (2019), were two artificial ground waterworks without disinfection located in the same city, two surface waterworks with UV-light disinfection combined with chlorine dioxide (ClO_2_) and chlorine (Cl_2_) or chloramine (NH_2_Cl) disinfection, and a ground waterworks with UV-light and chlorine (NaOCl) disinfection (systems A-E; Table 1]). Large-volume (100 liter) samples of cold drinking water after flushing of 1–2 min and hot water without flushing were collected over the four seasons of the year, twice in each season, using the dead-end ultrafiltration method (Smith and Hill, 2009). If the water contained chlorine, 1 g of sodium thiosulphate (Na_2_S_2_O_3_ x 5H_2_O) dissolved in 125 ml of sample water was added to the ultrafiltration cartridge to prevent residual disinfection after sampling. Cold water sampling points were stationed at three different distances (Table 1) from the waterworks in the same distribution line in each DWDS, except for sampling point three in DWDS E, which was on a different line in the same distribution system. Hot water samples were collected only at point two in all five systems. Building type and used pipe material in sampling points are in Supplemental Table S1.

### 2.2. Biofilm sampling

Additionally, biofilm samples using pipe collectors and loose deposit with mature biofilms of water meters were collected from the DWDSs as described in Inkinen et al. (2019). Three one-year-old pipe collectors were collected from each of distribution systems D and E, for a total of six. Pipe collectors were located at point two of the water sampling points. Also, a total of ten mature biofilm/deposit samples aged 6 to 19 years old were collected from the same DWDSs as water samples but mainly from different points of the pipelines. Large-volume water samples were taken from the same sampling points as mature biofilms of water meters (Table 1).

### 2.3. Water quality analyses

Water quality analyses including heterotrophic plate count (HPC), total bacteria cell count determined using 4,6-diamidino-2-phenylindole staining (DAPI), and physicochemical water parameters, including measurements of free and total chlorine, pH, temperature, oxygen, and electric conductivity in the field and in the laboratory, metal concentrations, and microbial nutrient levels were determined as described earlier by Ikonen et al., 2017.

### 2.4. Nucleic acid extraction, sequencing, and PCR

Dead-end ultrafiltration eluates were further concentrated by filtering the samples through 0.45-μm-pore-size, 47-mm-diameter polycarbonate filters (pore size 0.4 μm, Nuclepore Polycarbonate, Whatman, Kent, UK) and freezing at −80 °C before extraction of nucleic acids as previously described (Inkinen et al., 2019). In brief, DNA and RNA were extracted using a Chemagen magnetic kit (Perkin Elmer, Waltham, MA, USA). RNA was further purified using an Ambion TURBO DNA-free DNase kit (Life Technologies, Carlsbad, CA, USA), and then the cDNA was synthesized using a random hexamer primed Superscript III system for RT-PCR (Thermo Fisher Scientific, Waltham, MA, USA). Total RNA was stored at −80 °C, while cDNA and DNA extracts were stored at −20 °C until use.

For high-throughput amplicon sequencing, the samples were barcoded and analysed using the Illumina Miseq platform (LGC Genomics GmbH, Berlin, Germany). The nucleic acids were used as templates for polymerase chain reaction amplification with the modified primer set 341F (5′-CCTACGGGNGGCWGCAG-3′) and 785R (5′-GACTACHVGGGTATCTAAKCC-3′) (Klindworth et al., 2013) (Supplemental Table S2) and targeting the V3-V4 hypervariable region of the 16S rRNA gene, together with Nextera DNA Library Kit adapters. For quality control, negative controls from sample processing steps of water and biofilm samples without template DNA and positive controls with mock microbial community were analysed. Final products were quantity and quality assessed on Qubit dsDNA (Life Technologies) and Bioanalyzer DNA (Agilent, USA) high-sensitivity assay kits, then stored at −20 °C before pooling for sequencing. Equimolar amounts of purified amplicons (100 ng) were pooled and used to construct Illumina libraries using the Ovation Rapid DR. Multiplex System 1–96 (NuGEN).

The occurrence and abundance of *Mycobacterium* spp., *Legionella* spp., *Pseudomonas* spp., and occurrence of species *Mycobacterium intracellulare, Mycobacterium avium, Legionella pneumophila, Legionella pneumophila* serogroup 1in samples were measured with RNA and DNA extracts (including extraction and filtration blanks). The TaqMan RT-qPCR assays were performed as previously described (Pitkänen et al., 2013). The qPCR reactions were performed using a QuantStudio 6 real-time PCR system (Applied Biosystems) on 20 μl volume using TaqMan Environmental PCR Master Mix (Life Technologies) with primers and probes at final concentrations of 0.2 μM (IDT Technologies, Inc.; for primer and probe sequences, see Supplemental Table S2). The cycling conditions included 95 °C for 10 min enzyme activation and pre-denaturation followed by 40 cycles of 95 °C for 15 s denaturation and 60 °C for 60 s annealing. Standard curves were generated using artificial gene fragments (gBlocks, IDT Technologies, Inc.) containing the sequences for each of the targeted genes.

In qPCR, undiluted and 10-diluted cDNA and DNA samples were used as qPCR templates to detect PCR polymerase inhibition. For samples in which PCR inhibition was detected, qPCR data was generated using the results from diluted samples. Background signals, if detected in filtration blanks, were subtracted from all the results to generate final qPCR data per assay. In most cases the limit of detection (LOD) was set at 3 copies per reaction. The copy number per 100 ml of water was calculated for those samples with values above the limit of quantification (LOQ) (i.e., as determined by the lowest value within the quantification range). The final RT-qPCR and qPCR, equivalent LOD (eLOD) and equivalent LOQ (eLOQ) values were calculated after taking into account the volume of sample filtered, factors associated with the various processing steps of the RNA and DNA manipulations, and the dilutions used for each sample analysed (Supplemental Table S3).

### 2.5. Next generation sequencing data preprocessing and analysis

Raw reads were used after primer removal as input in Cutadapt software (Martin, 2011) to remove the Illumina TruSeq adapters.Reads without adapters were further quality trimmed with Trimmomatic software (Bolger et al., 2014). Then the forward and reverse reads were merged using Flash2 software (Magoč and Salzberg, 2011). The merged reads were quality processed using split_libraries_fastq.py script on the QIIME platform and demultiplexed (Caporaso et al., 2010). The reads were checked for chimeras (artificial sequences due to PCR error) using vsearch algorithm and chimeric sequences were filtered out. After chimera removal, the reads were clustered at 97 % using uclust_ref and an operational taxonomic unit (OTU) picking step was performed with an open reference OTU picking approach. Further downstream processing was performed by removal of chloroplast and mitochondrial OTUs (Inkinen et al., 2019). Sequence processing of the samples included negative (*N* = 41) and positive (*N* = 9) controls. Negative controls of sample processing were used to define a minimum read count for a sample to be included in the analysis and the count was 918 sequences. Samples with less than that were excluded. Taxonomy of sequences was obtained using database GTDB R207 (released in April 2022). The datasets generated during the study including bacteria sequences are available in the Short Read Archive (SRA) of NCBI (https://www.ncbi.nlm.nih.gov/) under BioProject PRJNA509718.

### 2.6. Phylogenetic analysis of opportunistic pathogens

Phylogenetic trees of *Legionella, Mycobacterium*, and *Pseudomonas* were constructed using reference strain sequences from a database, sequence libraries from the DWDSs studied, and an outgroup (*Coxiella burnetii* for *Legionella, Nocardia asteroides* for *Mycobacterium*, and *Acinetobacter calcoaceticus* for *Pseudomonas*). The 16S rRNA gene sequences of clinically relevant *Legionella, Mycobacterium*, and *Pseudomonas* species were collected from the NCBI nucleotide database (https://www.ncbi.nlm.nih.gov/nucleotide) for reference data. The corresponding sequences with the same OTU IDs were picked from the sequence libraries studied based on the Greengenes database using the closed reference approach. The picked sequences were aligned to the 16S rRNA gene region by mothur using align.seqs command with gg_13_8_99.align and were trimmed for equal length. All the sequences were aligned using ClustalW and used for the phylogenetic tree construction by Neighbour Joining algorithm using 1000 bootstrap values in MEGA5.2.

### 2.7. Statistical analyses

Statistical calculations for alpha diversities were performed using Chao1, Shannon, and Simpson indices. The alpha diversities (microbial community differences within samples) were calculated using alpha_rarefaction.py script with QIIME. Beta diversities (microbial community differences between the samples) and the summary of taxonomy in samples were calculated using MicrobiomeAnalyst. Before statistical analysis RNA and DNA sequence libraries were filtered in MicrobiomeAnalyst using default settings to remove low count sequences and normalized using default settings with total sum scaling. Beta diversities were visualized using Bray-Curtis dissimilarity index. Pairwise comparison between sample groups from different DWDSs and sampling points were calculated using permutational multivariate analysis of variance (PERMANOVA). Microsoft Excel was additionally used for preparing figures. Further statistical comparison of diversity and abundance of active members and opportunistic pathogens in different sample groups were calculated using IBM SPSS Statistics. Canonical correspondence analysis (CCA) was used to show relations between bacterial communities in DWDSs A-E and physico-chemical parameters, i.e., temperature, pH, turbidity, absorbance 254 nm and 420 nm, electric conductivity (EC), aluminum (Al), copper (Cu), iron (Fe), manganese (Mn) concentration, chlorine, and microbially available nutrients, i.e., acetate carbon, assimilable organic carbon (AOC), and microbially available phosphorus (MAP); as well as between community composition and microbiological water quality parameters, i.e., total bacteria cell count (DAPI) and heterotrophic plate count (HPC).

## 3. Results and discussion

### 3.1. The structural differences in the bacterial communities

A total of 4 255 115 sequencing reads and 5 836 OTUs were identified as bacteria after removing samples with sequence counts of less than 918 (Supplemental Table S4). More than two sequence reads were recorded for 5 183 identified OTUs. Average and maximum read count per sample were 12 441 and 58 206 sequences, respectively.

**Fig. 1.**
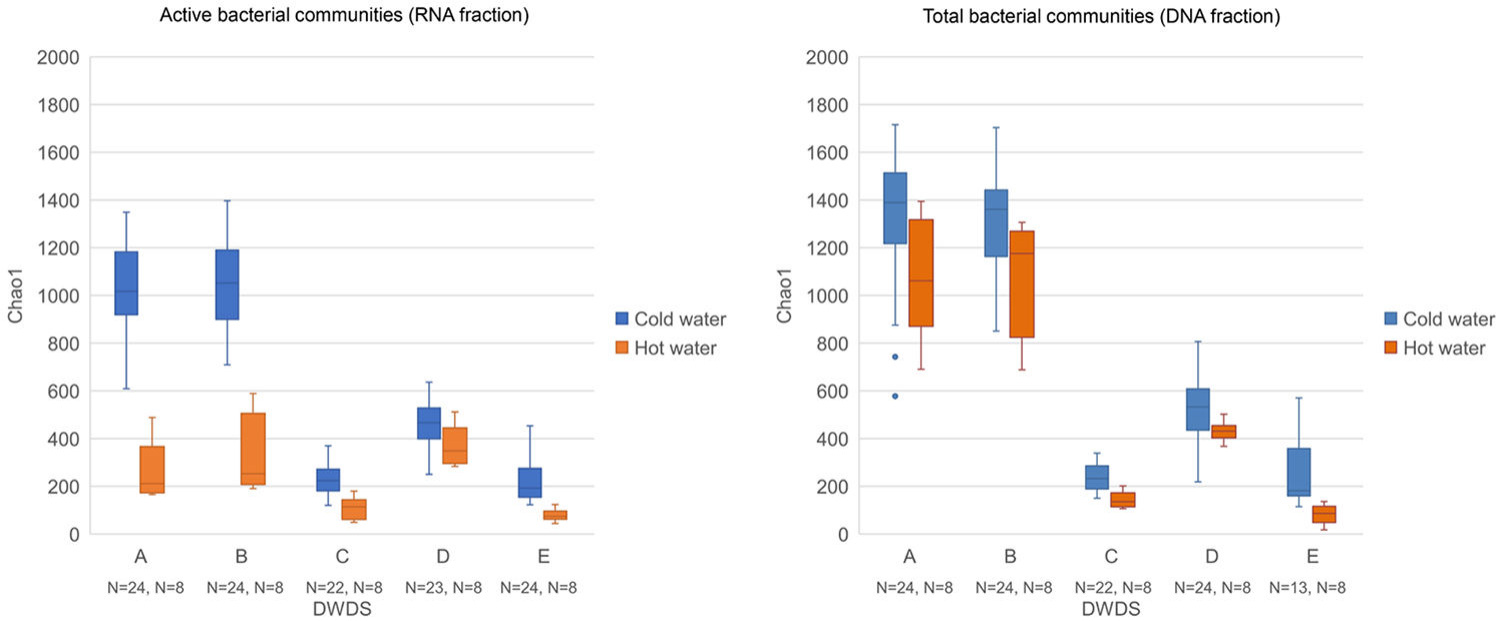
Alpha diversity of active (RNA fraction) and total (DNA fraction) bacterial communities by Chao1 index in cold and hot water in DWDSs A-E. Circles are outlier results.

**Fig. 2.**
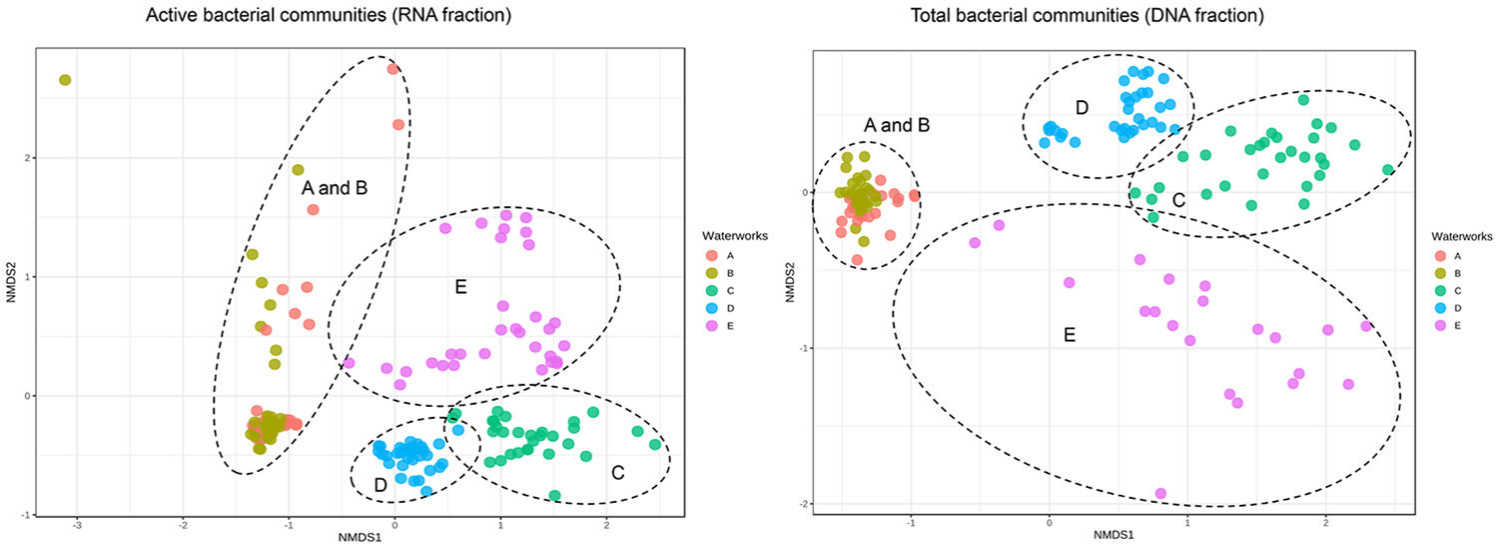
Non-metric multidimensional scaling (NMDS) plots of beta diversity of bacterial community compositions of RNA and DNA water samples using Bray-Curtis dissimilarity index in DWDSs A-E.

Species richness of active bacterial community (RNA fraction) was significantly higher in the cold water samples from the two non-disinfected DWDSs using artificially recharged groundwater (Chao1 diversity index mean values of 1 029; *N* = 24 and 1 042; *N* = 24 at DWDS A and B, respectively) as compared to the samples from the three disinfected DWDSs (Chao1 mean values 232; *N* = 22 at DWDS C, 471; *N* = 23 at D, and 219; *N* = 24 at E) (Kruskal-Wallis pairwise comparison, *P* < 0.05) (Fig. 1), similar to the findings of earlier genome-based studies (Bautista-de los Santos et al., 2016; Dai et al., 2020). Additionally, species richness was significantly higher in chloraminated DWDS D, compared to the two DWDSs (C and E) that used free chlorine compounds for chlorination (Kruskal-Wallis pairwise comparison, *P* < 0.05). In total bacteria communities (DNA fraction), differences of bacterial diversity between non-disinfected and disinfected DWDSs were similar but diversity in non-disinfected DWDSs was even higher compared to disinfected (Fig. 1). Source water and other treatment methods varied between the DWDSs and they may also have contributed to the differences. The difference in active bacterial community structures (RNA samples) between non-disinfected artificial ground waterworks and disinfected groundwater and surface water was clear in the beta-diversity analyses (Fig. 2). The DWDS bacteria from non-chlorinated artificial ground waterworks (A and B) were closer to each other compared to DWDS bacteria in chlorinated groundwater (E) or in surface waterworks (C and D). Similar clustering was also observed based on DNA samples. Furthermore, early studies of these DWDSs found a higher diversity of eukaryotic and archaea communities in non-disinfected DWDSs (A-B) compared to disinfected systems (C-E) (Inkinen et al., 2019 and 2021). In contrast, the abundance of functional genes based on metagenomic analysis was higher in disinfected DWDSs samples than in non-disinfected samples (Gomez-Alvarez et al., 2023).

**Fig. 3.**
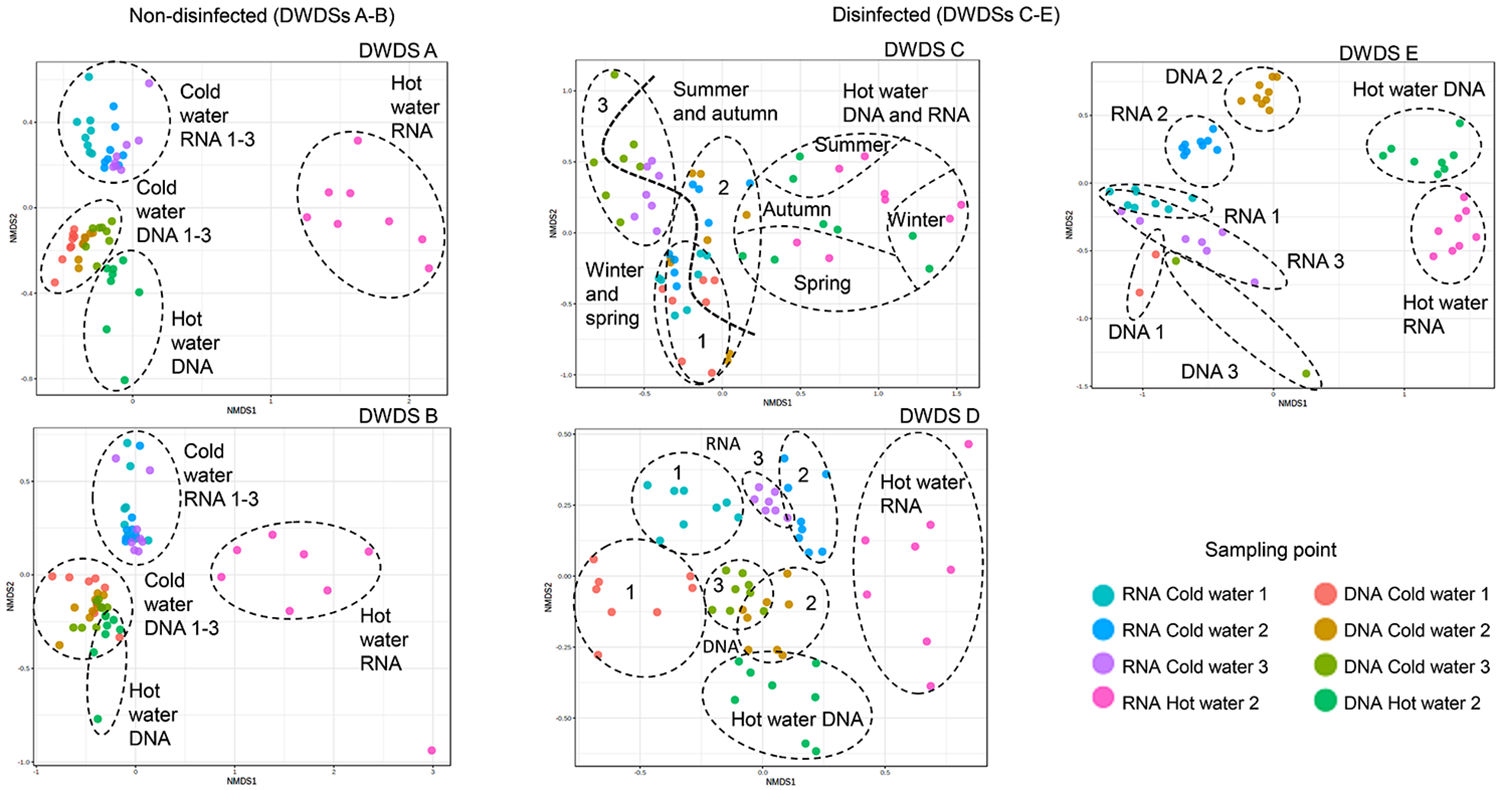
Non-metric multidimensional scaling (NMDS) plots of beta diversity of bacterial community compositions measured using Bray-Curtis dissimilarity index in each five studied drinking water distribution systems A-E. Samples from three sampling points (1–3) in cold water distribution systems and hot water samples from sampling point 2 are indicated by different colors.

Significantly higher bacterial community richness was noted in cold water samples (Chao1 mean 696; *N* = 224) than in samples from hot water systems (Chao1 mean 394; *N* = 80) (Mann-Whitney U test, *P* < 0.05). The higher alpha diversity in cold water compared to hot water samples was seen more strongly in non-disinfected DWDSs than in disinfected (Fig. 1). When comparing samples only from sampling point two, where hot water samples were collected, a significant difference in alpha diversity (Kruskal-Wallis pairwise comparison, *P* < 0.05) were noted in DWDSs A (non-disinfected artificial groundwater) between cold (Chao1 mean 1 091; *N* = 8) and hot water (264; *N* = 8) as well as in DWDS B between cold (Chao1 mean 1 112; *N* = 8) and hot water (322; *N* = 8) in active RNA fraction. In DWDS E (disinfected groundwater) in sampling point 2 difference was noted in both RNA fraction between cold (Chao1 mean 312; *N* = 9) and hot water (82; *N* = 8)) and in DNA fraction between cold (Chao1 mean 303; *N* = 9) and hot water (79; *N* = 8) (Kruskal-Wallis pairwise comparison, *P* < 0.05). The active part (RNA fraction) of the bacterial community was less diverse compared to the total DNA community. For comparison, most of the published research of bacterial communities have used DNA-based methods (Potgieter et al., 2018; Bruno et al., 2018; Waak et al., 2019). In cold water, significantly lower species richness was noted when bacterial communities were analysed according to RNA fraction (Chao1 mean 606; *N* = 117) (Mann-Whitney U test, *P* < 0.05) compared to DNA fraction (Chao1 mean 794; *N* = 107). Also, in hot water systems, species richness was significantly lower in RNA fraction (Chao1 mean 229; *N* = 40) than in DNA fraction (Chao1 mean 559; *N* = 40) (Mann-Whitney U test, *P* < 0.05). Similar differences between the sample groups were also seen when alpha diversity was measured as Shannon and Simpson indexes (Supplemental Figure S1). However, in DWDS A, cold water sampling point 3 showed a less diverse bacterial community than in hot water system (measured as Chao1 index). Furthermore, in DWDS C, the alpha diversity of the bacterial community in cold water at point 3, measured as Shannon and Simpson indexes, was less diverse than in hot water. Water temperature (cold vs. hot water system) shaped the bacterial community composition in each DWDSs. This was also seen in beta-diversity analysis (Fig. 3). However, in DWDSs B and D the water temperature was a less dominant factor as compared to the difference between groups of active bacteria (RNA) and total bacteria (DNA). Total bacterial communities in drinking water distribution systems based on DNA methods have been more often described in publications (Potgieter et al., 2018; Bruno et al., 2018; Waak et al., 2019; Rosenqvist et al., 2023) compared to hot water systems and active RNA-based communities. Earlier studies show that raising the temperature in hot water systems shifts bacterial composition (Ji et al., 2017; Dai et al., 2018) and bacterial communities are less diverse in hot tap water than in cold tap water (Zhang et al., 2021).

Changes in the bacterial community structure between the sampling points 1–3 inside each distribution system were also noted in beta-diversity analysis (Fig. 3). The biggest difference was between the first and the third sampling point in disinfected DWDS C (Bray-Curtis Index, DNA: R^2^=0.60, RNA: R^2^=0.48) and DWDS D (DNA: R^2^=0.56, RNA: R^2^=0.43) (*P* < 0.005, PERMANOVA). In DWDS E, points 1 and 3 clustered closely to each other, but sampling point 2 differed from sampling point 1 (RNA: R^2^=0.50, *P* = 0.001, PERMANOVA). In non-disinfected DWDSs A and B not as clear separation of sampling points was seen in NMDS figure (Fig. 3), but the sample group from DWDS A cold water sampling point 3 was separated from point 1 by Bray-Curtis dissimilarity index (DNA: R^2^=0.49, RNA: R^2^=0.53, *P* = 0.001, PERMANOVA). Chlorine concentrations may explain separation in DWDS E as they were higher at sampling points 1 and 3 as compared to sampling point 2 (Table 1). Aging of water, i.e. the distance from the water treatment plant has been noted to change the bacterial community structure especially in chloraminated DWDS by Potgieter et al. (2018), and to change the eukaryotic and archaea communities in the DWDSs studied here (Inkinen et al., 2019 and 2021). The effect of season on structure of bacterial communities was limited. In DWDS C it had some effect on beta diversity of community structure (Bray-Curtis Index, RNA: R^2^=0.31–0.38, DNA: R^2^=0.15–0.26, *P* < 0.005, PERMANOVA) and alpha diversity as it was higher in spring (Chao1 mean 277; *N* = 16) than in summer (185; *N* = 16) and autumn (172, *N* = 12) (Kruskal-Wallis, *P* < 0.05) but not in winter (Chao1 mean 252; *N* = 16). Also, in DWDS B season affected only a little on beta diversity (RNA: R^2^=0.22–0.31, DNA: R^2^=0.25–0.29, *P* < 0.005, PERMANOVA).

Biofilms from water meters (Chao1 mean 383; *N* = 18, Supplemental Table S5.) and pipe collectors (Chao1 mean 172; *N* = 11) show significantly less diversity (Kruskal-Wallis test, *P* < 0.05) than water samples (Kruskal-Wallis test, *P* < 0.05). The exception was DWDS E where the Chao1 indexes of the two water meters were higher (Chao1 mean 408; *N* = 5) than those of the water samples (cold water: Chao1 mean 233; *N* = 37, and hot water: Chao1 mean 80; *N* = 16). Less diverse communities had earlier been observed in biofilms compared to water samples, and more clearly in chloraminated water than in water without disinfectant residues (Waak et al., 2019). Bacterial communities of biofilm samples of DWDSs A-E clustered separately in beta-diversity analysis (Supplemental Figure S2). Additionally, young biofilms collected from pipe collectors clustered separately from mature biofilms of water meters (Supplemental Figure S2). A similar clustering of biofilm samples separate from water samples was seen in eukaryotic and archaea communities in the same DWDSs in earlier studies (Inkinen et al., 2019 and 2021).

The two most abundant bacteria in the active part of the community (RNA) and the total bacterial community (DNA) in water samples belonged to classes *Alphaproteobacteria* (RNA 28 %, DNA 44 %) and *Gammaprotebacteria* (RNA 32 %, DNA 30 %). The third most abundant and active bacteria class was *Vampirovibrionia* (formerly *Melainabacteria*) (15 %), whereas in total community (DNA) the third most abundant class was *Paceibacteria* (11 %). The *Alpha*- and *Gammaproteobacteria*, and *Vampirovibrionia* (formerly *Melainabacteria*) had earlier been found in drinking water environments using DNA-based methods (Bruno et al., 2018; Zhang et al., 2021), and they were active in these DWDSs based on RNA fraction.

**Fig. 4.**
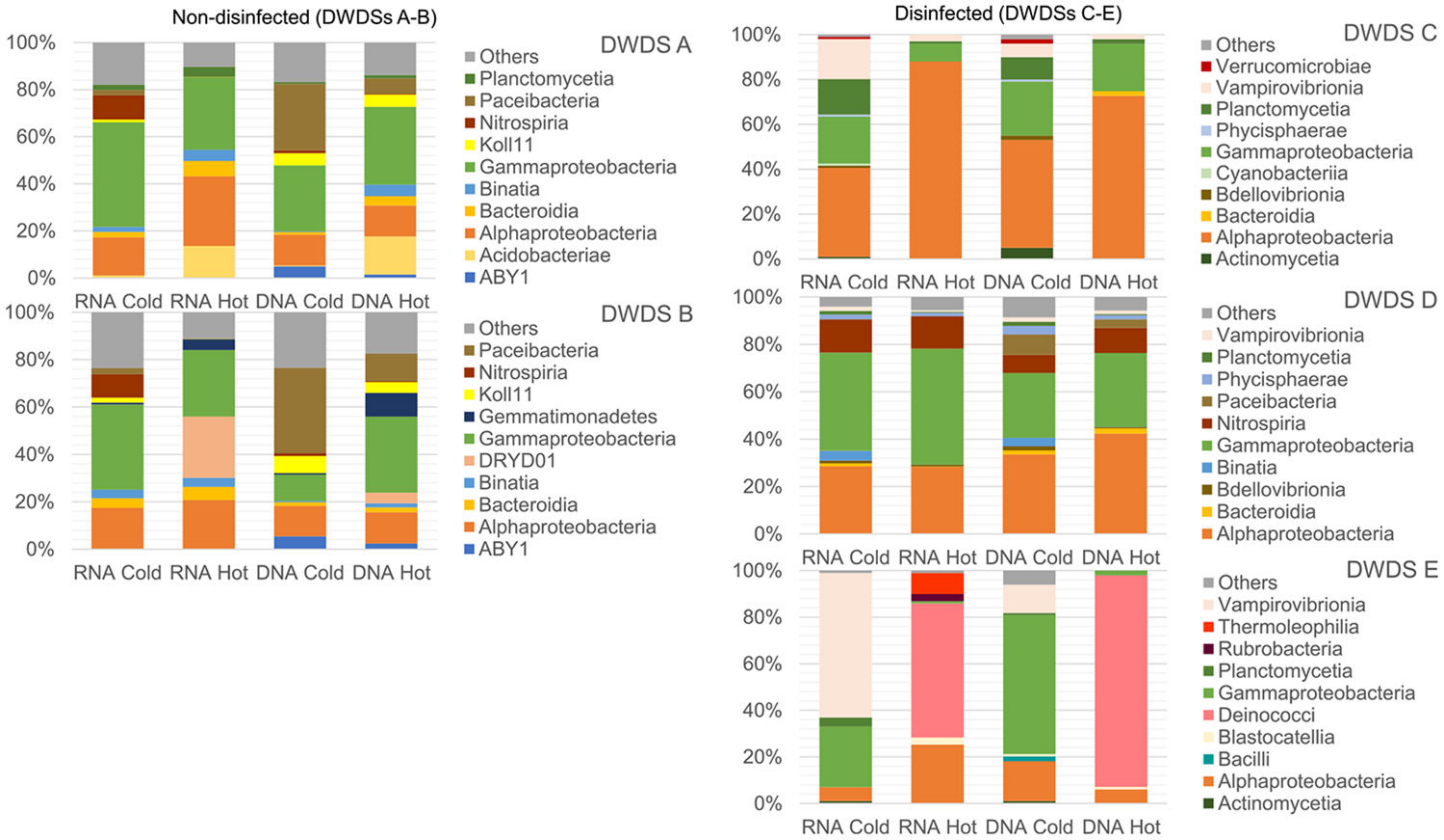
The ten most abundant bacterial classes in the studied drinking water distribution systems A-E in RNA and DNA fractions in cold water and hot water samples. A group called Others consist of the minor bacterial classes other than the top ten.

Non-disinfected groundwater DWDSs A and B had seven classes in common in the top ten most abundant bacterial classes in water samples (Fig. 4). Abundance of unassigned bacteria (group koll 11) and minor groups other than the top ten most abundant classes were higher in the waters in non-disinfected DWDSs A and B. *Vampirovibrionia* was seen in all disinfected DWDSs C, D, E, but was not in the top ten in non-disinfected DWDSs A and B. The relative abundance of *Vampirovibronia* was highest in cold water in DWDS E for both RNA and DNA fractions. *Vampirovibrionia* was also an active member (RNA fraction) in biofilm samples collected from water meters from DWDS E (Supplemental Figure S3). Class *Nitrospiria* was more abundant and active in cold and hot water in DWDS D with chloramine disinfection, compared to non-chlorinated DWDSs A and B that had *Nitrospiria* only in cold water and in greater abundance in active RNA fraction than in DNA fraction (Fig. 4). In the biofilm samples, *Nitrospiria* was the most abundant in DWDS D but was also active in the other DWDSs, except DWDS C (Supplemental Figure S3). *Nitrospiria* plays a role in nitrification in the bacterial ecosystem as it oxidizes nitrite into nitrate. The nitrification process may decrease water quality, cause corrosion, decrease disinfectant residue, and increase growth of bacteria (Hossain et al., 2022). *Bdellovibrionia* was active in cold water in chlorinated DWDS C and in cold and hot water in chloraminated water in DWDS D. Some species of *Bdellovibrio* (such as *B. bacteriovorus* and *B. exovorus*) belonging to *Bdellovibrionia* have been found to be natural predators of Gram-negative human pathogens and have also been found in chloraminated drinking water and in disinfection residue-free DWDS (Bautista-de los Santos et al., 2016; Atterbury and Tyson, 2021; Rosenqvist et al., 2023). In hot water systems, *Acidobacteriae* were present and active in DWDS A and *Deinococci* in DWDS E. *Acidobacteriae* have been found in hot water system studies (Ji et al., 2017). *Deinococci* class has been found to increase in temperatures as high as 51 °C in water systems (Dai et al., 2018). Also, *Blastocatellia, Rubrobacteria*, and *Thermoleophilia* were abundant active members of the bacterial community in the hot water in DWDS E. These bacteria have been observed in cold and hot tap water based on DNA (Zhang et al., 2021). Other bacteria detected in this study – *Cyanobacteria, Nitrospiria, Paceibacteria*, and *Planctomycetia* – have also been found in drinking water (Bruno et al., 2018; Dai et al., 2020; Rosenqvist et al., 2023). However, *Paceibacteria*, belonging to phylum *Patescibacteria*, were not as abundant active members of DWDSs A, B, and D, being found more in the DNA fraction.

**Fig. 5.**
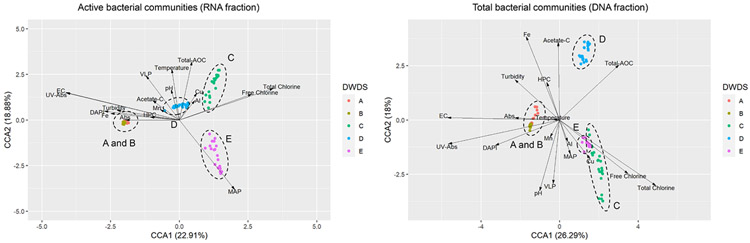
Canonical correspondence analysis (CCA) showing relationships between active RNA fraction and total DNA fraction of bacterial communities (colored dots) and physico-chemical and microbiological parameters of cold water samples in DWDSs A-E. Arrows are for temperature, pH, turbidity, absorbance 254 nm (UV-Abs) and 420 nm (Abs), electric conductivity (EC), aluminum (Al), copper (Cu), iron (Fe), manganese (Mn), free and total chlorine, assimilable organic carbon (AOC), acetate carbon, microbially available phosphorus (MAP), total bacteria cell count (DAPI), heterotrophic plate count (HPC), and virus-like particle (VLP).

Environmental factors of cold water correlate with the bacterial communities of DWDSs differently, as seen in canonical correspondence analysis (Fig. 5). Iron (Fe) correlated more with non-disinfected artificial groundwater DWDSs A-B compared to disinfected DWDSs C-E. Copper (Cu) and aluminum (Al) correlated with DWDSs C-D using disinfected water originating from surface waterworks based on CCA. A previous study found a higher abundance of metal resistance genes in disinfected than in non-disinfected systems (Tiwari et al., 2022). Microbially assimilable phosphorus (MAP) correlated with DWDS E (disinfected groundwater supply). However, in DNA fraction these chemical parameters do not correlate as strongly with bacterial community results (Fig. 5).

### 3.2. Activity of opportunistic pathogens in cold and hot water systems

Of the opportunistic pathogens, 22 OTUs of *Legionella*, 16 of *Mycobacterium* and eight of *Pseudomonas* were detected in total from all five DWDSs using closed reference OTU picking comprising 0.9 % of all the bacteria from water and biofilm samples total in RNA and DNA fractions. Additionally, 16 OTUs of *Legionella*, 14 of *Mycobacterium*, and two of *Pseudomonas* were detected using de novo picking. All OTUs of opportunistic pathogens that were detected in biofilm samples were present in water samples as well, but not all bacteria present in water samples were detected in biofilm samples. The (RT)-qPCR confirmed the presence of opportunistic pathogens in the five DWDSs studied as *Legionella* spp. was detected in 85 %, *Mycobacterium* spp. in 95 %, and *Pseudomonas aeruginosa* in 78 % of the water and biofilm samples.

**Fig. 6.**
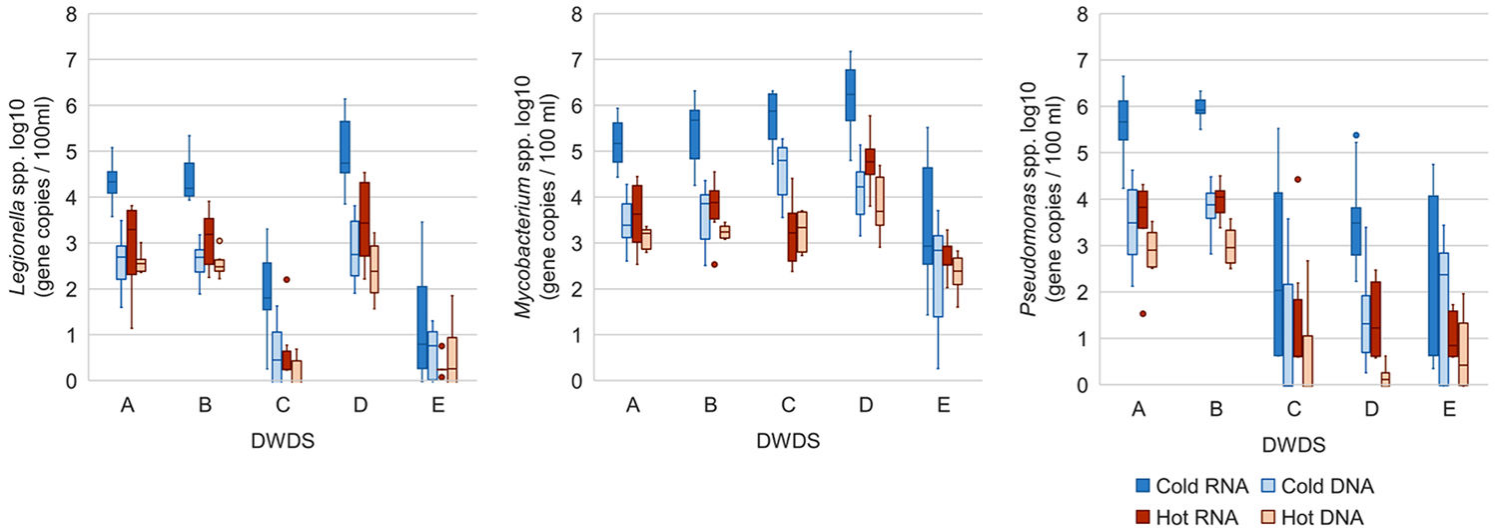
*Legionella, Mycobacterium*, and *Pseudomonas* genera in cold and hot water samples in RNA and DNA fractions in each DWDS A-E analysed using (RT-)qPCR method (gene copies / 100 ml) at logarithmic scale. Circles are outliers.

In cold water samples, *Legionella* spp. was significantly more abundant in RNA fraction (mean 201 gene copies / 100 ml, *N* = 117) compared to DNA fraction (mean 127 gene copies / 100 ml, *N* = 107) (Kruskal-Wallis test, *P* < 0.001) measured by RT-qPCR and qPCR methods, respectively. In RNA fraction, the abundance of *Legionella* was significantly higher in cold water than in hot water (mean RNA 131 gene copies / 100 ml, *N* = 40) (Kruskal-Wallis test, *P* < 0.001), indicating that the hot water temperature was efficient in controlling the activity of *Legionella* at the sampling locations studied. This difference was seen in all DWDSs A-E but the difference was not so clear at DWDS E (disinfected groundwater) (Fig. 6). The mean temperature of the hot water samples was above 50 °C, which may explain the lower abundance of opportunistic pathogens, including *Legionella*, which decreases at such high temperatures (Dai et al., 2018). To control the growth of *Legionella* in hot water, the temperature should be kept above 50 °C (preferably 55 °C) (ESGLI, 2017).

Like *Legionella, Mycobacterium* spp. was significantly more abundant in RNA fraction in cold water (mean 219 gene copies/100 ml, *N* = 117) compared to hot water samples (mean 110 gene copies/100 ml, *N* = 40) (Kruskal-Wallis test, *P* < 0.001). In DNA fraction, a difference between cold water (mean 124 gene copies/100 ml, *N* = 107) and hot water (mean 75 gene copies/100 ml, *N* = 40) was also detected (Kruskal-Wallis test, *P* = 0.005). A difference in abundance of *Mycobacterium* between cold and hot water was observed in all DWDSs A-E (Fig. 6). High hot water temperatures here may cause the lower abundance compared to cold water, as described with *Legionella*.

In cold water samples, *Pseudomonas* spp. was significantly more abundant in RNA fraction (mean 201 gene copies/100 ml, *N* = 117) compared to DNA fraction (mean 134 gene copies/100 ml, *N* = 107) (Kruskal-Wallis test, *P* < 0.001). Also, *Pseudomonas* abundance was significantly higher in cold water than in hot water (mean RNA 129 gene copies/100 ml, *N* = 40) but only in RNA fraction (Kruskal-Wallis test, *P* < 0.001), indicating that *Pseudomonas* was more active in cold water compared to hot water: as with *Legionella*, this was probably caused by high water temperatures. This was seen in all DWDSs A-E but not as clearly in disinfected systems C-E (Fig. 6). There were small but not significant differences in *Pseudomonas* spp. numbers between hot water DNA (mean 83 gene copies/100 ml, *N* = 40) and RNA with higher RNA numbers, as well as between cold and hot water in DNA samples with higher numbers in cold water.

The detection frequency of opportunistic pathogens was higher in RNA fraction than in DNA fraction in most cases, when (RT)-qPCR method targeted at the opportunistic pathogen species was used (Fig. 6). No such difference in detection frequency was seen when an amplicon sequencing method targeted at the 16S RNA gene was used (Supplemental Figure S6). This might be because amplicon sequencing is not a quantitative method but produces the relative abundance of members in the bacterial community (Bautista-de los Santos et al., 2016). Opportunistic pathogens detected with the RNA method more likely describe the living cells, since RNA is synthesized by active cells and RNA degrades faster than DNA does (Pitkänen et al., 2013; Li et al., 2017). Living cells may possibly start growing under favorable conditions, causing health risks for consumers.

### 3.3. Abundance of opportunistic pathogens changed between DWDSs

In RNA fraction in cold water, *Pseudomonas* spp. were most abundant in non-disinfected DWDSs A (mean 7.8 × 10^5^ gene copies/100 ml, *N* = 24) and B (mean 1.1 × 10^6^ gene copies/100 ml, *N* = 24) based on the (RT)-qPCR method (Fig. 6). However, waterworks with disinfection did not necessarily have lower numbers of opportunistic pathogens than waterworks without disinfection. DWDS D, a chloramine using surface water supply, had the highest gene copy numbers of *Legionella* spp. (mean 2.4 × 10^5^ gene copies/ml, *N* = 23) and *Mycobacterium* spp. (mean 3.3 × 10^6^ gene copies/100 ml, *N* = 23).

Although numbers of *Legionella* were highest in chloraminated DWDS D they were lowest in DWDSs C (mean 326 gene copies/100 ml, *N* = 22) and E (mean 168 gene copies/100 ml, *N* = 23), with free chlorine disinfection. In contrast, earlier studies have found chloramine to be more effective against *Legionella* than chlorine disinfection (Donohue et al., 2019; Gomez-Alvarez et al., 2012). Additionally, *Legionella pneumophila* was found in two samples from location 2 in non-disinfected DWDS A in cold water and from location 3 in cold water in non-disinfected DWDS B, as well as in the water sample collected from the water meter sampling location in DWDS B (Table 2). *Legionella pneumophila* serogroup 1 was found only in DWDS A from location 2 in four cold water samples and in one hot water sample.

Ten of the detected *Legionella* OTUs were determined to belong to genus *Legionella* by QIIME software (circles in phylogenetic tree, Supplemental Figure S5) and 12 of the OTUs were identified by QIIME only at family level, belonging to family *Legionellaceae* and confirmed as *Legionella* using NCBI BLAST (triangles in the phylogenetic tree). The most abundant OTU in water samples (OTU ID 3479378) had more RNA reads (5 196) than DNA reads (1 032). The second most abundant *Legionella* (OTU ID 311942) had 669 RNA and 710 DNA reads. Both of these OTUs were close to many *Legionella* sequences in NCBI database but did not match any *Legionella* species with 100 % similarity. Other *Legionella* OTUs had read counts of less than one thousand. *Legionella pneumophila* is the most well-known pathogenic *Legionella* species, but other *Legionella* species may cause infections as well (Chauhan and Shames, 2021).

Mycobacteria were most abundant at DWDSs C and D, which distribute disinfected water from surface water works. Mycobacteria are known to be more resistant to many disinfection chemicals, including chlorine, than other bacteria (Bautista-de los Santos et al., 2016; Gomez-Alvarez et al., 2012). Similarly, Kotlarz et al. (2019) observed higher numbers of mycobacteria in drinking water that used surface water. DWDSs C and D had nutrient AOC corresponding with bacterial communities in canonical correspondence analysis (CCA, Fig. 5). Mycobacteria prevalence is found to correlate strongly with the concentration of assimilable organic carbon in the water leaving the waterworks (Torvinen et al., 2004). Opportunistic pathogens may, however, grow and cause a health risk even in water with low nutrient contents (Vital et al., 2012; Wang et al., 2013), and they were also found in this study in low nutrient ground water and artificial ground water. *Mycobacterium avium* was found in cold water samples in RNA fraction in DWDSs A and B. *M. avium* was found at locations 1 and 2 in DWDS A, and at locations 2 and 3 in DWDS B (Table 2). *M. avium* was also found in cold water samples gathered from the same location as water meter samples. *Mycobacterium intracellulare* was found in only one DNA water meter biofilm sample showing that opportunistic pathogen species may be present in biofilms even though not detected in water.

One of the *Mycobacterium* OTUs (OTU ID 1062748) detected was close to *M. fortuitum* and another one (OTU ID 2651333) was close (87 %) to *M. haemophilum* (Supplemental Figure S6). The three most abundant OTUs in the water samples were OTU 543570 with 1 598 RNA and 5 170 DNA reads, OTU 1062748 (close to *M. fortuitum*) with 3 371 RNA and 2 194 DNA reads, and OTU 902334 with 1 065 RNA and 123 DNA reads. Other closed-reference picked *Mycobacterium* OTUs had less than one thousand read counts. Additionally, there were two de novo picked *Mycobacterium* OTUs with read counts of over one thousand.

*Pseudomonas* species were most abundant in non-disinfected DWDSs A and B, which distribute artificial groundwater. The most abundant OTU belonging to genus *Pseudomonas* in the water samples was OTU 288207, with a total of 3 691 reads, which was not close to any known opportunistic pathogen in phylogenetic analysis (Supplemental Figure S7). *Legionella* spp., *Mycobacterium* spp., and *Pseudomonas* spp. were also present in RNA and DNA fractions in biofilm or loose deposit samples (Supplemental Table S6) in five DWDSs A-E.

This study shows that there are opportunistic pathogens in non-disinfected and disinfected drinking water and biofilms in active (RNA) fraction and total (DNA) fraction, and their abundance varies between DWDSs with different disinfection strategies showing that opportunistic pathogens are also present in water distribution systems of good quality. Potential health risks exist if the water quality changes so that it provides an opportunity for pathogen bacteria to multiply, i.e. if the water temperature rises in cold water or falls in hot water.

## 4. Conclusions

- Not all members of the communities in cold and hot water systems were active as active RNA fraction was less diverse than DNA fraction of total bacterial community.
- Active communities in the studied good quality drinking waters were more diverse in non-disinfected water distribution systems compared to chloraminated systems. The least diverse communities were in systems where free chlorine was used as a disinfectant.
- The structure of active bacterial communities varied between cold and hot water systems. *Nitrospiria* was active in non-disinfected system only in cold water but in chloraminated systems both in cold and hot water. *Deinococci* were active in hot water in chlorinated groundwater systems and in non-disinfected hot water systems.
- Beta diversity of bacterial communities changed between the sampling points in distribution systems, representing the age of the water within each DWDS, and more in disinfected systems with disinfectant residue than in non-disinfected systems.
- Opportunistic pathogens were detected in cold and hot water systems with qPCR method. *Legionella* and *Mycobacterium* genera were most abundant in chloraminated and *Pseudomonas* in non-disinfected systems, but their abundance using amplicon sequencing was <1 % of all bacteria.

## Supplementary Material

Supplemental Information

## Figures and Tables

**Table 1:** Drinking water distribution systems (DWDSs) A-E and their sampling locations. Samples from cold and hot water sampling points, young pipe biofilm (Pipe) and matured water meter biofilm samples, distance from the drinking water treatment plant (DWTP), average and standard deviation of free and total chlorine concentrations and temperature. Additional 100 l cold water samples collected with water meter biofilms: N(DWDS A) = 2, N(DWDS B) = 2, and N(DWDS C) = 1. na = not applicable.

DWDS		Sampling point	Distance from DWTP (km)	Sample type	RNA samples	DNA samples	Free Cl (mg/l) mean ± sd	Total Cl (mg/l) mean ± sd	Temperature (°C) mean ± sd
No disinfection	A	1	2	Cold water	8	8	na	na	12.7 ± 4.3
		2	8	Cold water	8	8	na	na	10.6 ± 3.0
		2	8	Hot water	8	8	na	na	53.8 ± 2.3
		3	11	Cold water	8	8	na	na	9.2 ± 3.4
		na	na	Water meter	2	2	na	na	na
	B	1	1	Cold water	8	8	na	na	9.5 ± 1.8
		2	3	Cold water	8	8	na	na	10.8 ± 1.8
		2	3	Hot water	8	8	na	na	57.5 ± 3.5
		3	8	Cold water	8	8	na	na	8.0 ± 2.1
		na	na	Water meter	2	2	na	na	na
Chlorine + UV	C	1	2	Cold water	7	7	0.41 ± 0.16	0.47 ± 0.12	8.9 ± 7.5
		2	8	Cold water	8	8	0.38 ± 0.30	0.33 ± 0.09	11.8 ± 5.0
		2	8	Hot water	8	8	na	na	50.6 ± 3.9
		3	20	Cold water	7	7	0.09 ± 0.10	0.16 ± 0.10	10.1 ± 4.3
		na	na	Water meter	2	1	na	na	na
Chloramine + UV	D	1	5	Cold water	8	8	0.06 ± 0.09	0.15 ± 0.06	10.3 ± 3.1
		2	14	Cold water	8	8	0.09 ± 0.10	0.12 ± 0.14	10.5 ± 4.0
		2	14	Hot water	8	8	na	na	54.6 ± 2.8
		3	19	Cold water	7	8	0.07 ± 0.08	0.14 ± 0.13	8.1 ± 3.2
		na	na	Water meter	1	1	na	na	na
		2	14	Pipe	3	3	na	na	na
Chlorine + UV	E	1	9	Cold water	8	2	0.19 ± 0.04	0.28 ± 0.11	5.7 ± 1.5
		2	26	Cold water	9	9	0.11 ± 0.05	0.12 ± 0.05	7.0 ± 2.1
		2	26	Hot water	8	8	na	na	54.1 ± 4.1
		3*	36	Cold water	7	2	0.21 ± 0.08	0.34 ± 0.21	5.1 ± 1.1
		na	na	Water meter	3	2	na	na	na
		2	26	Pipe	3	2	na	na	na

* Sampling point 3 is collected from another pipeline than points 1 and 2.

**Table 2:** Number of samples (N) where opportunistic pathogen species from genera *Legionella* and *Mycobacterium* were detected in DWDSs A-E by quantitative PCR method. ND=Not detected, WM=water meter. For (RT)-qPCR assay details, see Supplemental Tables S2 and S3.

	*Legionella pneumophila* (studied from the DNA fraction)			*Mycobacterium* species (studied by 16S rRNA and 16S rRNA gene assays)		
DWDS	N (total)	*L. pneumophila* detected	*L. pneumophila* serogroup 1 detected	N (total)	*M. avium* detected	*M. intracellulare* detected
A	38	2 (cold water)	4 (cold water) 1 (hot water)	75	6 (RNA, cold water)	ND
B	38	2 (cold water)	ND	76	4 (RNA, cold water)	ND
C	33	ND	ND	67	ND	ND
D	36	ND	ND	70	ND	ND
E	25	ND	ND	63	ND	1 (DNA, biofilm WM)

## Data Availability

The bacteria sequence data generated in this study is available in the Short Read Archive (SRA) of NCBI (https://www.ncbi.nlm.nih.gov/) under BioProject PRJNA509718.
