# Supplementary material for: Composition of active bacterial communities and presence of opportunistic pathogens in disinfected and non-disinfected drinking water distribution systems in Finland
Source: Water Res. Author manuscript; Available in PMC 2024 Feb 5. (PMC10840642; doi:10.1016/j.watres.2023.120858)
Supplement: Supplemental Information [file NIHMS1961004-supplement-Supplemental_Information.docx]

Supplementary Materials

Composition of active bacterial communities and presence of opportunistic pathogens in disinfected and non-disinfected drinking water distribution systems in Finland

**Sallamaari Siponen ^a,b,^*, Balamuralikrishna Jayaprakash^a,1^, Anna-Maria Hokajärvi^a^, Vicente Gomez-Alvarez^c^, Jenni Inkinen^d, 2^, Ivan Ryzhikov^b^, Pia Räsänen^a^, Jenni Ikonen^a^, Anna Pursiainen^a^, Ari Kauppinen^a,1^, Mikko Kolehmainen^b^, Jussi Paananen^d^, Eila Torvinen^b^, Ilkka T. Miettinen^a^, Tarja Pitkänen^a,e,^***

*^a^Finnish Institute for Health and Welfare, Department of Health Security, P.O. Box 95, 70701 Kuopio, Finland*

*^b^University of Eastern Finland, Department of Environmental and Biological Sciences, P.O. Box 1627, 70211 Kuopio, Finland*

*^c^U.S. Environmental Protection Agency, Office of Research and Development, 26W. Martin Luther King Dr., Cincinnati, OH 45268, United States*

*^d^University of Eastern Finland, Institute of Biomedicine, P.O. Box 1627, 70211 Kuopio, Finland*

*^e^University of Helsinki, Faculty of Veterinary Medicine, Department Food Hygiene and Environmental Health, P.O. Box 66, 00014 Helsinki, Finland, Finland*

^1^ Present address: Finnish Food Authority, Laboratory and Research Division, Animal Health Diagnostic Unit, Helsinki, Finland

^2^ Present address: Biohit Oyj, Helsinki, Finland.

***Corresponding Authors

*Finnish Institute for Health and Welfare, Department of Health Security, FI-70701 Kuopio, Finland.

E-mail address: sallamaari.siponen@thl.fi. Phone: 358 29 524-6514.

*Finnish Institute for Health and Welfare, Department of Health Security, FI-70701 Kuopio, Finland.

E-mail address: tarja.pitkanen@thl.fi. Phone: 358 29 524-6315.

**Supplemental Tables and Figures**

**Supplemental Table S1.** Water source, building where cold and hot water samples from a tap or biofilm samples from pipe collector and water meter was collected, and pipe material used in the building. na=data not available.

|  |  |  |  | | |  | |  |  | |  |
| --- | --- | --- | --- | --- | --- | --- | --- | --- | --- | --- | --- |
| DWDS | | Source water | | Sampling point | Building | | Pipe material | | | Sample type | |
| No disinfection | A | Artificial groundwater | | 1 | Office (water purification plant) | | Cast iron + plastic | | | Cold water | |
|  |  |  |  | 2 | Office building | | Cast iron + plastic | | | Cold and hot water | |
|  |  |  |  | 3 | Water pumping station | | Cast iron + plastic | | | Cold water | |
|  |  |  |  | na | Office and storage buildings | | na | | | Water meter biofilm | |
|  | B | Artificial groundwater | | 1 | Day care center | | Cast iron + plastic | | | Cold water | |
|  |  |  |  | 2 | School | | Cast iron + plastic | | | Cold and hot water | |
|  |  |  |  | 3 | Care home | | Cast iron + plastic | | | Cold water | |
|  |  |  |  | na | Day care center and grocery store | | na | | | Water meter biofilm | |
| Chlorine + UV | C | Surface water | | 1 | Water pumping station | | Asbestos cement | | | Cold water | |
|  |  |  |  | 2 | Office building | | Segmented cast iron + plastic | | | Cold and hot water | |
|  |  |  |  | 3 | Health care center | | Cast iron + plastic | | | Cold water | |
|  |  |  |  | na | Office building | | Cast iron + plastic | | | Water meter biofilm | |
| Chloramine + UV | D | Surface water | | 1 | Office building | | Steel / cast iron | | | Cold water | |
|  |  |  |  | 2 | Office building | | Steel / asbestos cement / cast iron | | | Cold and hot water | |
|  |  |  |  | 3 | Water pumping station | | Steel / asbestos cement / cast iron | | | Cold water | |
|  |  |  |  | na | Gas station | | na | | | Water meter biofilm | |
|  |  |  |  | 2 | Office building | | Steel / asbestos cement / cast iron | | | Pipe biofilm | |
| Chlorine + UV | E | Groundwater | | 1 | Pressure station of water tower | | Plastic | | | Cold water | |
|  |  |  |  | 2 | Office (waterworks) | | Asbestos cement | | | Cold and hot water | |
|  |  |  |  | 3 | Water tower | | Plastic | | | Cold water | |
|  |  |  |  | na | na | | na | | | Water meter biofilm | |
|  |  |  |  | 2 | Water station | | Asbestos cement | | | Pipe biofilm | |

**Supplemental Table S****2.** The oligonucleotide sequences used in the high-throughput sequencing and (RT)-qPCR assays in the study.

| **Assay name** | **Target species** | **Primers and probes** | **Length (bp)** | **Reference** |
| --- | --- | --- | --- | --- |
| High throughput sequencing: 16S rRNA gene | Bacteria | 341F: CCTACGGGNGGCWGCAG  785R: GACTACHVGGGTATCTAAKCC | 444 | Klindworth *et al.* (2013) and Herlemann *et al.* (2000) |
| Lssp Assay | *Legionella* spp. | Lspp F: TGGGGAGGAGGGTTGATA  Lspp R: CGGAGTTAGCCGGTGCTT  Lspp P: FAM-CTGTGGGTAACGTCCAGTTAATCAG | 73 | In-house method |
| Lmip Assay | *Legionella pneumophila* | Lmip_ESGLI F: GAAGCAATGGCTAAAGGCATGC  Lmip_ESGLI R: GAACGTCTTTCATTTGYTGTTCGG  Lmip_ESGLI P: FAM- CGCTATGAGTGGCGCTCAATTGGCTTTA -BHQ1 | 79 | Mentasti *et al.* (2015) |
| Lpne_sg1 Assay | *Legionella pneumophila*, serogroup 1 | Lpne sg1 F: CAAACACCCCAACCGTAATCA  Lpne sg1 R: CAAAGGGCGTTACAGTCAAACC  Lpne sg1 P: 5′-FAM- TCTTGGGATTGGGTTGGGTTATTTTAACTCCT -TAMRA | 75 | Mérault *et al*. (2011) |
| Mycobacteria assay | *Mycobacterium* spp. | Mf939: GATGCAACGCGAAGAACCTT  Mr1011: TGCACCACCTGCACACAGG  Mp960: FAM-CCTGGGTTTGACATGCACAGGACG-TAMRA | 91 | Räsänen et al. (2013), Torvinen et al. (2010) |
| MI Assay | *Mycobacterium intracellulare* | MAI_Fn: GGGTGAGTAACACGTGKGCAA  MI_Rn: CCACCTAAAGACATGCGMCTAAA  MAI_P: TGCACTTCGGGATAAGCCTGGGAAA | 100 | Chern *et al.* (2015) |
| M. avium assay | *Mycobacterium avium* | MAI_Fn: GGGTGAGTAACACGTGKGCAA  AviumRn: CCAGAAGACATGCGTCKTGA  MAI_P: TGCACTTCGGGATAAGCCTGGGAAA | 97 | Chern *et al.* (2015) |
| Pseudomonas (aeruginosa) Assay | *Pseudomonas* spp. | PsAerF: CAAAACTACTGAGCTAGAGTACG′  PsAerR: GCCACTGGTGTTCCTTCCTA  PsAerP: FAM- TCCTGTGTAGCGGTGAAATGCGTAGA-BHQ1 (Black Hole Quencher 1) | 89 | Matsuda *et al.* (2007) |

**Supplemental Table S3.** Limits of detection (eLOD), limits of quantitation (eLOQ), amplification efficiencies, and R^2^ of qPCR methods.

|  | Assay name | Target species | eLOD, copies  100 ml^-1^ (min-max) | eLOQ, copies  100 ml^-1^ (min-max) | (RT)-qPCR amplification efficiencies (min – max) | R^2^ (min-max) |
| --- | --- | --- | --- | --- | --- | --- |
| Cold and hot water samples | Lssp Assay | *Legionella* spp. | DNA: 0.6 - 21  RNA: 1.9 - 67 | DNA: 3.9 - 139  RNA: 13 - 448 | DNA: 79.4 - 99.3 RNA: 78.9 - 111.5 | DNA: 0.992 - 0.997 RNA: 0.969 - 0.999 |
|  | Lmip Assay | *Legionella pneumophila* | DNA: 2.0 - 69 | DNA: 4.5 - 159 | DNA: 87.8 - 95.6 | DNA: 0.992 - 0.998 |
|  | Lpne_sg1 Assay | *L. pneumophila*, serogroup 1 | DNA: 0.6 - 21 | DNA: 1 - 35 | DNA: 98.4 - 100.8 | DNA: 0.969 - 0.987 |
|  | Mycobacteria assay | *Mycobacterium* spp. | DNA: 0.79 - 28  RNA: 367 - 12 914 | DNA: 1 - 42  RNA: 368 - 12 959 | DNA: 90.9 - 106.3 RNA: 87.9 - 108.9 | DNA: 0.984 - 0.998 RNA: 0.967 - 0.998 |
|  | MI Assay | *Mycobacterium intracellulare* | DNA: 0.6 - 21  RNA: 1.9 - 67 | DNA: 1 - 35  RNA: 3 - 112 | DNA: 92.5 - 100.9 RNA: 95.5 - 118.1 | DNA: 0.981 - 0.998 RNA: 0.972 - 0.996 |
|  | M. avium assay | *Mycobacterium avium* | DNA: 0.6 - 21  RNA: 1.9 - 67 | DNA: 3.9 - 139  RNA: 13 - 448 | DNA: 83.8 - 99.7 RNA: 76.3 - 92.4 | DNA: 0.986 - 0.997 RNA: 0.984 - 0.999 |
|  | Pseudomonas (aeruginosa) Assay | *Pseudomonas* spp. | DNA: 0.98 - 35  RNA: 4.5 - 157 | DNA: 3.9 -139  RNA: 13 - 448 | DNA: 95.8 - 114.8 RNA: 87.8 - 103.8 | DNA: 0.952 - 1.000 RNA: 0.979 - 0.999 |
| Biofilm samples | Lssp Assay | *Legionella* spp. | DNA: 79 - 1 500  RNA: 256 - 4 856 | DNA: 526 - 10 000  RNA: 1 704 -32 375 | DNA: 79.4 - 99.3  RNA: 89.6 - 111.5 | DNA: 0.992 - 0.995  RNA: 0.974 - 0.998 |
|  | Lmip Assay | *Legionella pneumophila* | DNA: 263 - 5 000 | DNA: 605 - 11 500 | DNA: 95.6 | DNA: 0.998 |
|  | Lpne_sg1 Assay | *L. pneumophila*, serogroup 1 | DNA: 70 - 1 500 | DNA: 132 - 2 500 | DNA: 98.4 | DNA: 0.987 |
|  | Mycobacteria assay | *Mycobacterium* spp. | DNA: 105 - 2 000 | DNA: 158 - 3 000 | DNA: 96.0 - 98. 2 RNA: 87.9 - 106.4 | DNA: 0.984 - 0.994 RNA: 0.987 - 0.990 |
|  | MI Assay | *Mycobacterium intracellulare* | DNA: 79 - 1 500  RNA: 256 - 4 856 | DNA: 132 - 2 500 RNA: 426 - 8093 | DNA: 100.9 RNA: 95.5 | DNA: 0.981 RNA: 0.996 |
|  | M. avium assay | *Mycobacterium avium* | DNA: 79 - 1 500  RNA: 256 - 4 856 | DNA: 526 - 10 000  RNA: 1704 - 32 375 | DNA: 99.8  RNA: 92.4 | DNA: 0.987  RNA: 0.994 |
|  | Pseudomonas (aeruginosa) Assay | *Pseudomonas* spp. | DNA: 132 - 2 500  RNA: 596 - 11 331 | DNA: 526 - 10 000  RNA: 1704 - 32 375 | DNA: 95.8 - 114.8  RNA: 96.7 - 103.8 | DNA: 0.952 - 0.989  RNA: 0.983 - 0.999 |

**Supplemantal Table S4.** Sequence counts and the number (N) of different operational taxonomic units (OTUs) in water and biofilm samples of DWDSs A-E. Cold=water from cold water pipe; Hot=water from warm water pipe; Pipe=biofilm from pipe collector; WM=biofilm/loose deposit from watermeter; na=data not available.

|  |  |  |  | Active bacteria (RNA fraction) | | |  | Total bacteria (DNA fraction) | | |
| --- | --- | --- | --- | --- | --- | --- | --- | --- | --- | --- |
|  | DWDS | Sampling point | Sample type | N | Reads  (mean± std.dev.) | OTUs  (mean± std.dev.) |  | N | Reads  (mean± std.dev.) | OTUs  (mean± std.dev.) |
| No disinfection | A | 1 | Cold | 8 | 6100 ± 3500 | 990 ± 310 |  | 8 | 6900 ± 3100 | 1400 ± 330 |
|  |  | 2 | Cold | 8 | 8300 ± 4400 | 1300 ± 380 |  | 8 | 10000 ± 7700 | 1700 ± 370 |
|  |  |  | Hot | 8 | 13000 ± 12000 | 310 ± 120 |  | 8 | 7800 ± 6100 | 1000 ± 370 |
|  |  | 3 | Cold | 8 | 11000 ± 4700 | 1200 ± 370 |  | 8 | 12000 ± 7400 | 1400 ± 440 |
|  |  | na | WM | 2 | 10000 ± 4100 | 500 ± 47 |  | 2 | 17000 ± 410 | 790 ± 93 |
|  | B | 1 | Cold | 8 | 11000 ± 4900 | 1300 ± 390 |  | 8 | 9200 ± 5200 | 1400 ± 450 |
|  |  | 2 | Cold | 8 | 7800 ± 3700 | 1300 ± 380 |  | 8 | 6800 ± 3700 | 1500 ± 490 |
|  |  |  | Hot | 8 | 12000 ± 9800 | 350 ± 190 |  | 8 | 13000 ± 11000 | 1400 ± 500 |
|  |  | 3 | Cold | 8 | 6400 ± 4000 | 1100 ± 430 |  | 8 | 6800 ± 4200 | 1400 ± 430 |
|  |  | na | WM | 2 | 11000 ± 11000 | 590 ± 270 |  | 2 | 4100 ± 1000 | 610 ± 120 |
| Chlorine + UV | C | 1 | Cold | 7 | 18000 ± 12000 | 380 ± 89 |  | 7 | 8600 ± 6000 | 330 ± 110 |
|  |  | 2 | Cold | 8 | 19000 ± 11000 | 360 ± 100 |  | 8 | 11000 ± 8800 | 270 ± 140 |
|  |  |  | Hot | 8 | 19000 ± 13000 | 170 ± 75 |  | 8 | 13000 ± 12000 | 210 ± 110 |
|  |  | 3 | Cold | 7 | 22000 ± 5000 | 470 ± 110 |  | 7 | 12000 ± 7400 | 330 ± 160 |
|  |  | na | WM | 2 | 12000 ± 12000 | 130 ± 38 |  | 1 | 29 000 | 130 |
| Chloramine + UV | D | 1 | Cold | 8 | 21000 ± 9300 | 800 ± 150 |  | 8 | 14000 ± 9800 | 640 ± 200 |
|  |  | 2 | Cold | 8 | 13000 ± 9500 | 610 ± 120 |  | 8 | 11000 ± 4600 | 680 ± 87 |
|  |  |  | Hot | 8 | 5500 ± 3800 | 350 ± 110 |  | 8 | 8400 ± 5700 | 520 ± 160 |
|  |  | 3 | Cold | 7 | 17000 ± 6600 | 870 ± 130 |  | 8 | 16000 ± 7400 | 1100 ± 160 |
|  |  | na | WM | 1 | 51 000 | 610 |  | 1 | 55 000 | 910 |
|  |  | 2 | Pipe | 3 | 20000 ± 9300 | 320 ± 63 |  | 3 | 18000 ± 13000 | 260 ± 66 |
| Chlorine + UV | E | 1 | Cold | 8 | 20000 ± 19000 | 220 ± 76 |  | 2 | 2600 ± 410 | 110 ± 16 |
|  |  | 2 | Cold | 9 | 20000 ± 9800 | 540 ± 180 |  | 9 | 7300 ± 4400 | 390 ± 270 |
|  |  |  | Hot | 8 | 10000 ± 5400 | 97 ± 18 |  | 8 | 19000 ± 14000 | 130 ± 30 |
|  |  | 3* | Cold | 7 | 16000 ± 21000 | 210 ± 67 |  | 2 | 3200 ± 2200 | 120 ± 64 |
|  |  | na | WM | 3 | 17000 ± 12000 | 570 ± 160 |  | 2 | 18000 ± 4900 | 680 ± 87 |
|  |  | 2 | Pipe | 3 | 8500 ± 2400 | 190 ± 160 |  | 2 | 1300 ± 560 | 59 ± 30 |
|  | A | WM location | Cold | 1 | 18 000 | 2 000 |  | 2 | 5 700 | 1 400 |
|  | B | WM location | Cold | 2 | 12000 ± 6900 | 1600 ± 450 |  | 2 | 9700 ± 360 | 1700 ± 22 |
|  | C | WM location | Cold | 1 | 8 000 | 270 |  | 1 | 2 300 | 160 |

**Supplemental Table S5.** Alpha diversity of bacterial community in biofilm samples illustrated as Chao1, Shannon, and Simpson indexes.

|  | DWDS | Sample type | Number of samples | Nucleic acid | N | Chao1 average | Shannon average | Simpson average |
| --- | --- | --- | --- | --- | --- | --- | --- | --- |
| Non-disinf. | A | Water meter | 2 | DNA | 2 | 450 ± 76 | 6.38 ± 0.38 | 0.959 ± 0.029 |
|  |  |  |  | RNA | 2 | 440 ± 190 | 6.10 ± 0.92 | 0.955 ± 0.029 |
|  | B | Water meter | 2 | DNA | 2 | 660 ± 89 | 7.53 ± 0.11 | 0.988 ± 0.004 |
|  |  |  |  | RNA | 2 | 450 ± 22 | 6.28 ± 0.04 | 0.955 ± 0.003 |
| Disinfected | C | Water meter | 2 | DNA | 1 | 65 | 1.87 | 0.490 |
|  |  |  |  | RNA | 2 | 140 ± 100 | 3.05 ± 2.34 | 0.639 ± 0.318 |
|  | D | Water meter | 1 | DNA | 1 | 290 | 5.88 | 0.965 |
|  |  |  |  | RNA | 1 | 230 | 4.91 | 0.935 |
|  |  | Pipe | 3 | DNA | 3 | 210 ± 13 | 3.12 ± 0.11 | 0.671 ± 0.013 |
|  |  |  |  | RNA | 3 | 180 ± 13 | 3.80 ± 0.26 | 0.793 ± 0.043 |
|  | E | Water meter | 3 | DNA | 2 | 380 ± 25 | 7.04 ± 0.02 | 0.983 ± 0.003 |
|  |  |  |  | RNA | 3 | 420 ± 53 | 6.58 ± 0.53 | 0.971 ± 0.020 |
|  |  | Pipe | 3 | DNA | 2 | 100 ± 34 | 2.73 ± 0.50 | 0.635 ± 0.134 |
|  |  |  |  | RNA | 3 | 170 ± 132 | 2.86 ± 1.08 | 0.642 ± 0.240 |

**Supplemental Table S6*.*** *Legionella, Mycobacterium*, and *Pseudomonas* genera in biofilm samples from pipe collectors (Pipe) and loose deposits of water meters (WM) from DWDSs A-E analysed using (RT-)qPCR method (gene copies (GC) / ml) and high-throughput amplicon sequencing method (reads). Additionally heterotrophic plate count (HPC) in colony forming unit (cfu) / ml and total bacteria cell count. NA= Not available, <eLOD = below limit of detection,<eLOQ = below limit of quantification

|  |  | RNA fraction | | | | | | DNA fraction | | | | | |  |  |
| --- | --- | --- | --- | --- | --- | --- | --- | --- | --- | --- | --- | --- | --- | --- | --- |
|  |  | *Legionella* | | *Mycobacterium* | | *Pseudomonas* | | *Legionella* | | *Mycobacterium* | | *Pseudomonas* | | HPC | Total cells |
| DWDS | Sample type | GC/ml | reads | GC/ml | reads | GC/ml | reads | GC/ml | reads | GC/ml | reads | GC/ml | reads | cfu/ml | cells/ml |
| A | WM | 37 000 | 0 | 41 000 | 0 | 8 100 | 0 | 2 800 | 6 | 2 700 | 0 | 130 | 0 | 10 | 10 300 000 |
| A | WM | 1 400 | 0 | 35 000 | 0 | 11 000 | 0 | 59 | 2 | 2 400 | 2 | 110 | 0 | 180 | 6 600 000 |
| B | WM | 22 | 0 | 3 800 | 6 | 990 | 0 | 2 | 0 | 3 000 | 1 | 44 | 1 | 19 000 | 5 800 000 |
| B | WM | 340 | 0 | 1 700 | 1 | 970 | 0 | 110 | 0 | 1 800 | 1 | 200 | 0 | 8 400 | 5 300 000 |
| C | WM | 800 | 19 | 250 | 14 | 3 | 0 | NA | NA | NA | NA | NA | NA | 11 000 | 4 000 000 |
| C | WM | 2 | 0 | 3 300 | 0 | 5 | 0 | 1 | 0 | 310 | 1 | 1 | 1 | 660 | 410 000 |
| D | Pipe | 490 | 11 | 47 000 | 8 | <eLOD | 0 | <eLOQ | 9 | 5 700 | 10 | <eLOD | 0 | 1 900 000 | 2 900 000 |
| D | Pipe | 2 000 | 3 | 185 000 | 0 | <eLOD | 0 | <eLOQ | 1 | 11 000 | 12 | <eLOD | 0 | 1 800 000 | 2 700 000 |
| D | Pipe | 2 700 | 0 | 248 000 | 11 | <eLOD | 0 | <eLOD | 1 | 8 100 | 28 | <eLOD | 0 | 850 000 | 2 100 000 |
| D | WM | 4 800 | 14 | 7 900 000 | 82 | 780 | 1 | 3 | 15 | 76 000 | 260 | 2 | 5 | 65 000 | 340 000 |
| E | Pipe | <eLOD | 31 | 12 000 000 | 2 600 | 22 000 | 28 | <eLOD | 0 | 49 000 | 160 | 1 000 | 3 | 130 000 | 250 000 |
| E | Pipe | <eLOD | 3 | 2 100 000 | 490 | 15 000 | 34 | <eLOD | 0 | 5 300 | 150 | <eLOD | 4 | 32 000 | 330 000 |
| E | Pipe | <eLOD | 13 | 1 500 000 | 1 700 | 6 500 | 51 | NA | NA | NA | NA | NA | NA | 34 000 | 16 000 |
| E | WM | 1 900 | 15 | 6 900 | 0 | 10 | 0 | 2 200 | 19 | 10 000 | 9 | 2 | 0 | 84 000 | 5 500 000 |
| E | WM | 17 000 | 7 | 2 900 000 | 8 | 13 | 0 | 2 500 | 10 | 310 000 | 6 | 3 | 0 | 420 000 | 9 200 000 |
| E | WM | 7 | 24 | 14 000 | 34 | 16 | 4 | NA | NA | NA | NA | NA | NA | 220 000 | 6 900 000 |

**Supplemental Figures**


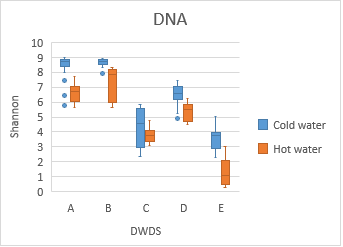

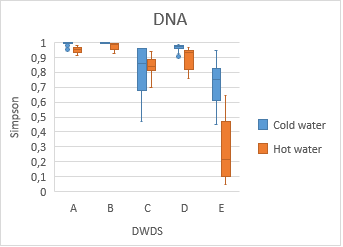

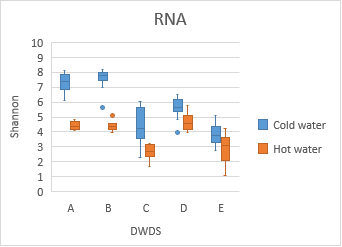

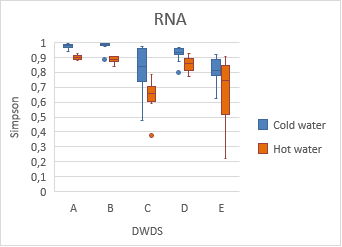


**Supplemental Figure S1.** Alpha diversity (Shannon, Simpson) in cold and hot water in DWDSs A-E in DNA and RNA fractions. Circles are outlier results.


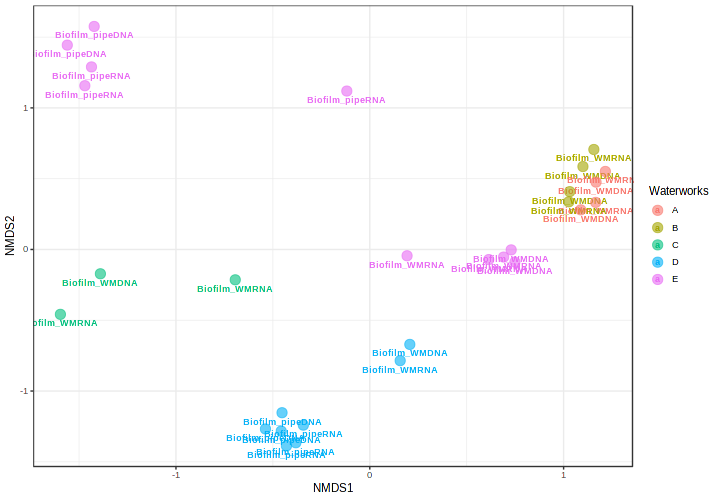


**Supplemental Figure S2.** Beta diversity in bacterial communities in biofilm samples in DWDSs A-E presented as a NMDS plot. PERMANOVA F-value: 6.2764; R-squared: 0.51126; p-value: 0.001 [NMDS] Stress = 0.11399.


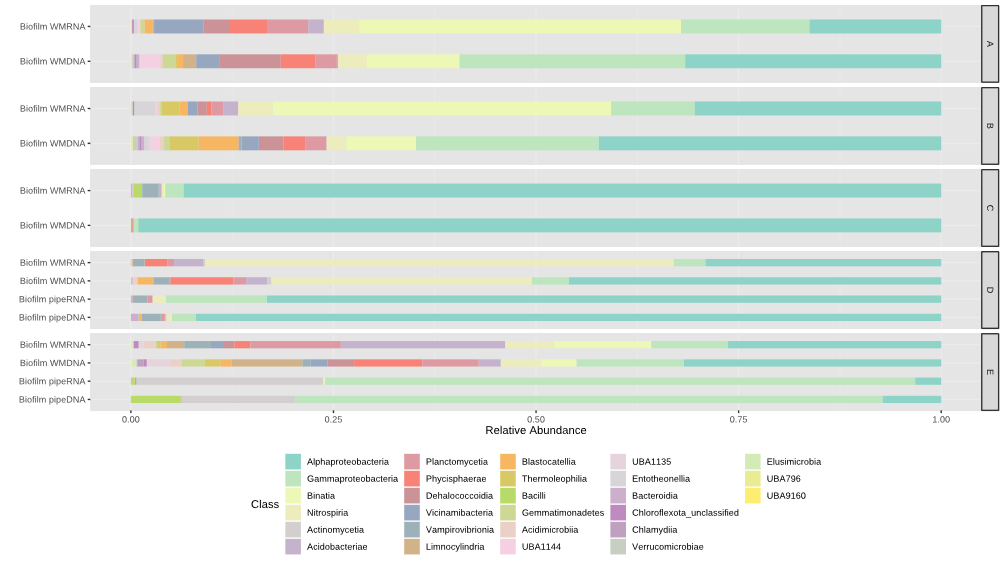


**Supplemental Figure S3.** Relative abundances of bacterial classes in the studied drinking water distribution systems A-E in groups of DNA and RNA fractions separately in biofilm samples. Biofilm WM = biofilm / loose deposit sample from water meter and biofilm pipe = biofilm of pipe collector.


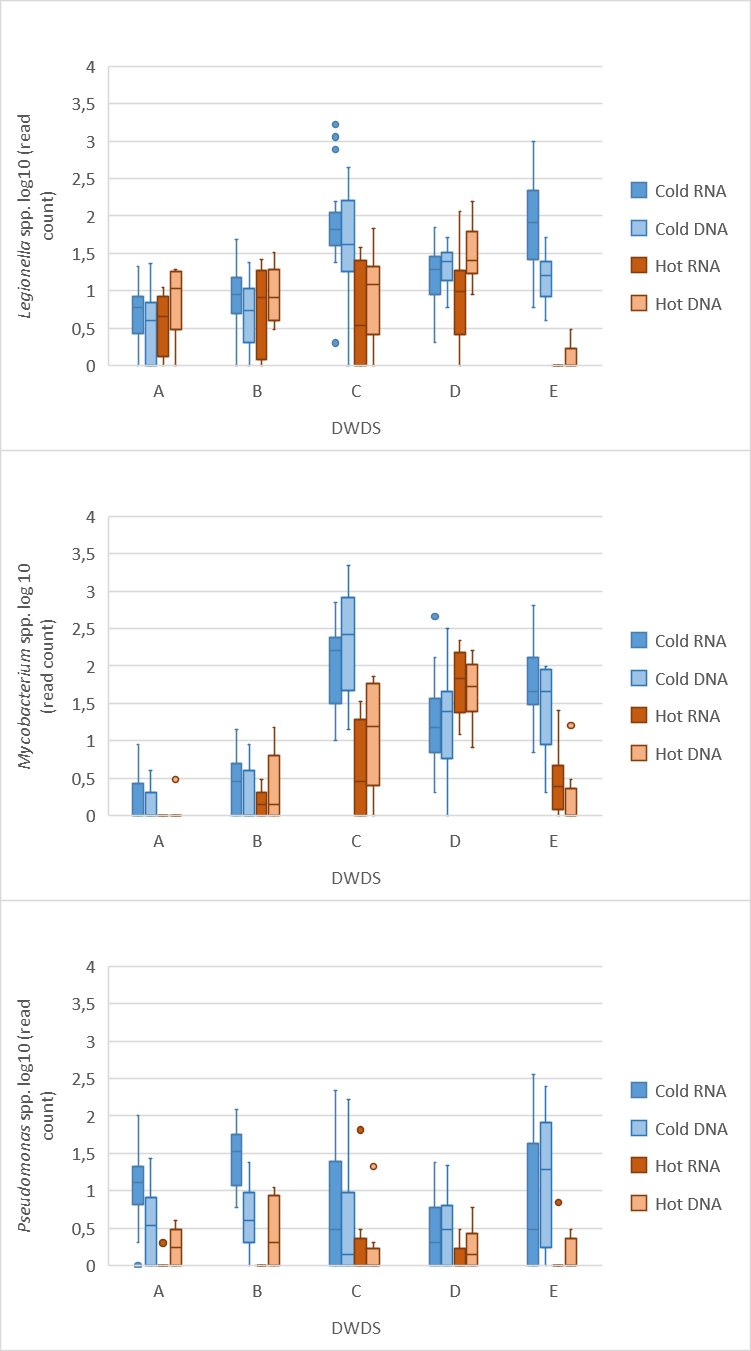
**Supplemental Figure S4.** *Legionella, Mycobacterium*, and *Pseudomonas* genera in cold and hot water samples in RNA and DNA fractions in each DWDS A-E analysed using amplicon sequencing method (reads) at logarithmic scale. Circles are outliers.


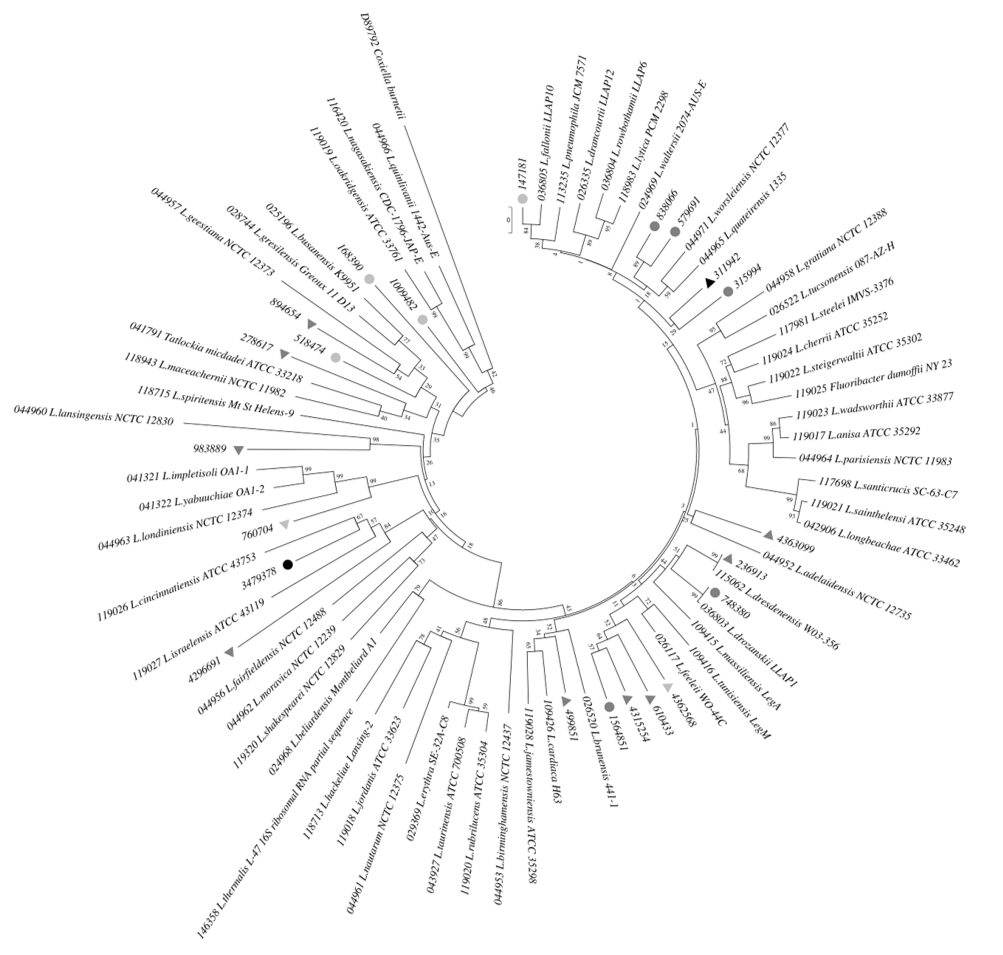


**Supplemental Figure S5.** Phylogenetic tree of *Legionella* OTUs detected in studied five drinking water distribution systems using closed reference picking. OTUs detected as *Legionella* at genus level are marked with circles and at family level and confirmed by comparing nucleotide database are marked with triangle. The darker the colour of the symbol the more abundant that OTU was (three categories <100; 100-1 000; >1000 sequence reads).

.


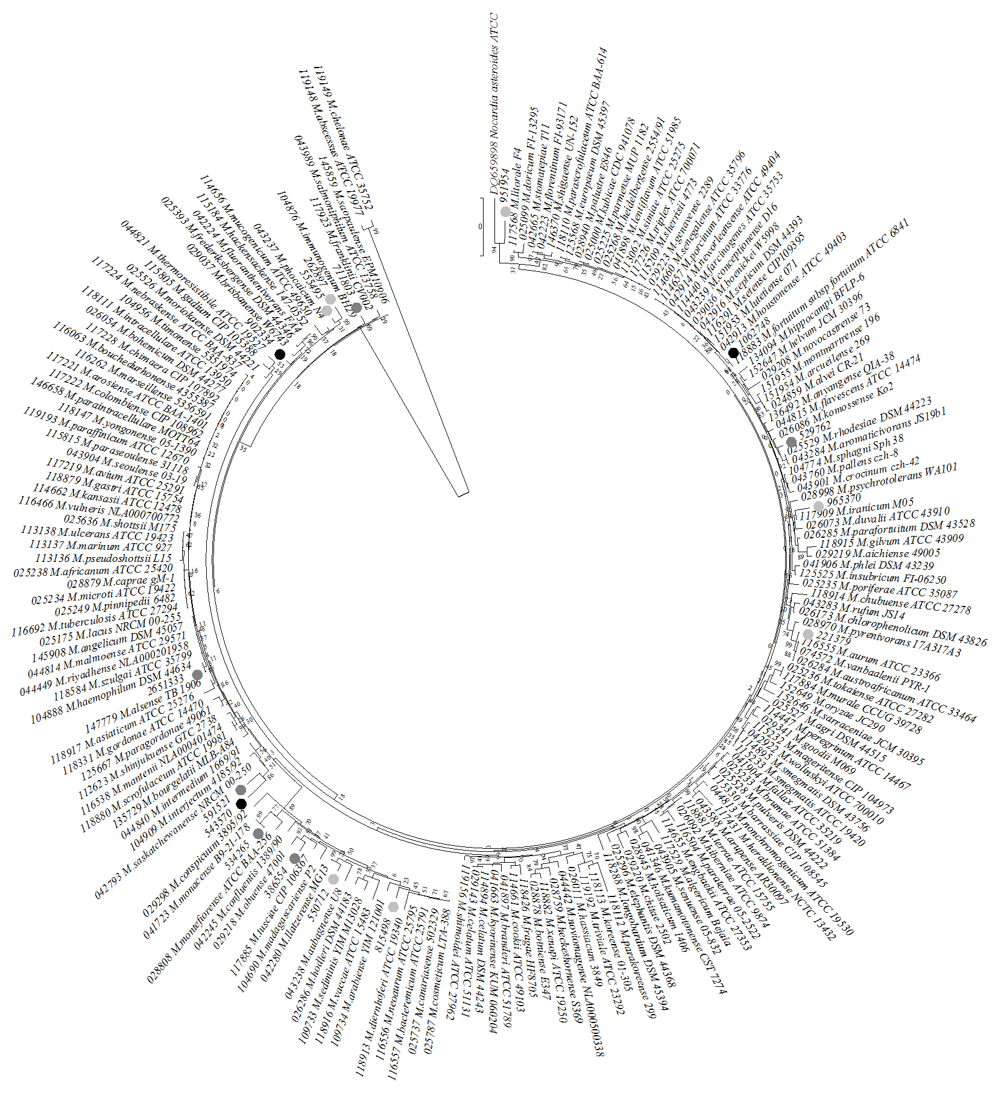


**Supplemental Figure S6.** Phylogenetic tree of *Mycobacterium* OTUs detected at genus level in studied five drinking water distribution systems using closed reference picking. The darker the colour of the symbol the more abundant that OTU was (three categories <100; 100-1 000; >1000 sequence reads).


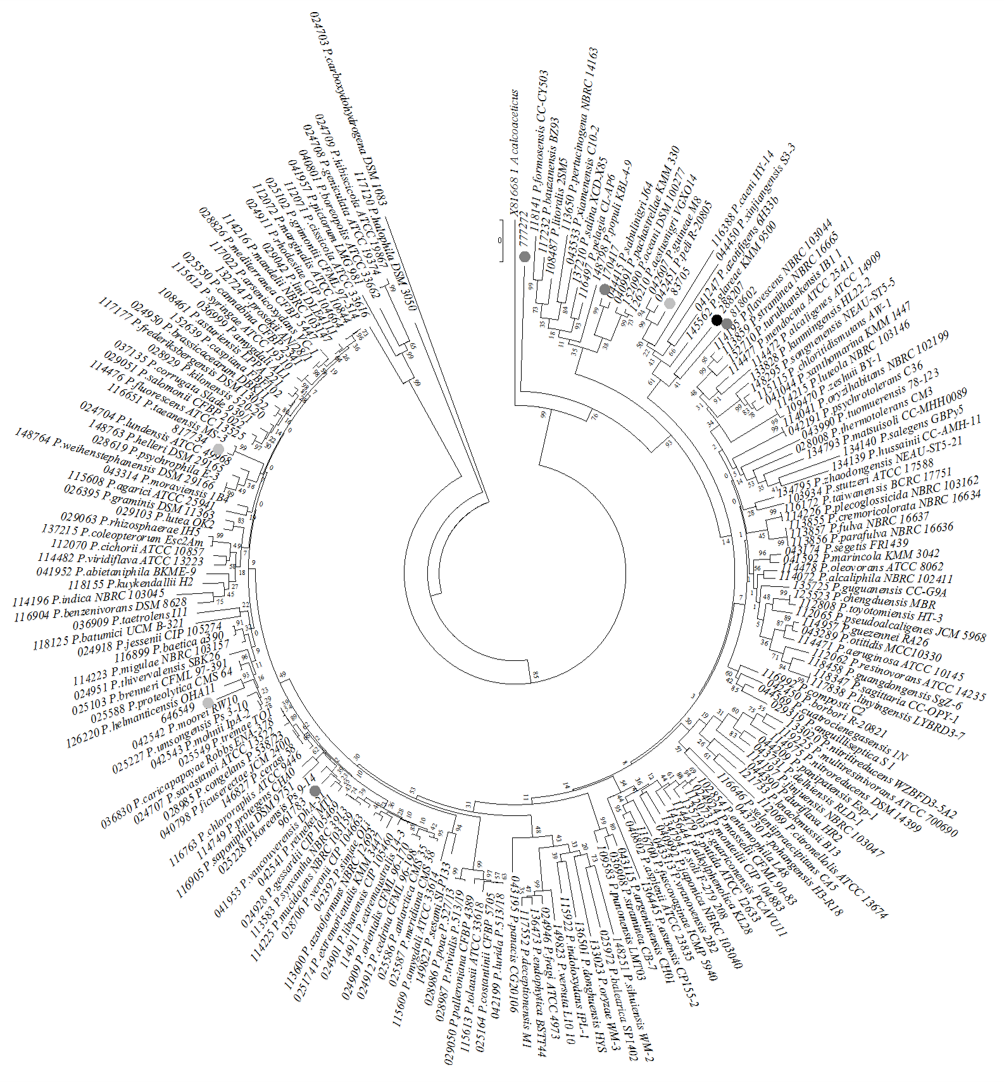


**Supplemental Figure S7.** Phylogenetic tree of *Pseudomonas* OTUs detected in studied five drinking water distribution systems using closed reference picking. The darker the colour of the symbol the more abundant that OTU was (three categories <100; 100-1 000; >1000 sequence reads).

**REFERENCES**

Chern EC, King D, Haugland R, Pfaller S. Evaluation of quantitative polymerase chain reaction assays targeting *Mycobacterium avium, M. intracellulare*, and *M. avium* subspecies *paratuberculosis* in drinking water biofilms. J Water Health. 2015 Mar;13(1):131-9. doi: 10.2166/wh.2014.060.

Herlemann DP, Labrenz M, Jürgens K, Bertilsson S, Waniek JJ, Andersson AF. Transitions in bacterial communities along the 2000 km salinity gradient of the Baltic Sea. ISME J. 2011 Oct;5(10):1571-9. doi: 10.1038/ismej.2011.41. Epub 2011 Apr 7. PMID: 21472016; PMCID: PMC3176514.

Klindworth A, Pruesse E, Schweer T, et al. Evaluation of general 16S ribosomal RNA gene PCR primers for classical and next-generation sequencing-based diversity studies. *Nucleic Acids Res*. 2013;41(1):e1. doi:10.1093/nar/gks808

Matsuda K, Tsuji H, Asahara T, Kado Y, Nomoto K. Sensitive quantitative detection of commensal bacteria by rRNA-targeted reverse transcription-PCR. Appl Environ Microbiol. 2007 Jan;73(1):32-9. doi: 10.1128/AEM.01224-06.

Mentasti M, Kese D, Echahidi F, Uldum SA, Afshar B, David S, Mrazek J, De Mendonça R, Harrison TG, Chalker VJ. Design and validation of a qPCR assay for accurate detection and initial serogrouping of *Legionella pneumophila* in clinical specimens by the ESCMID Study Group for Legionella Infections (ESGLI). Eur J Clin Microbiol Infect Dis. 2015 Jul;34(7):1387-93. doi: 10.1007/s10096-015-2363-4.

Mérault N, Rusniok C, Jarraud S, Gomez-Valero L, Cazalet C, Marin M, Brachet E, Aegerter P, Gaillard JL, Etienne J, Herrmann JL; DELPH-I Study Group; Lawrence C, Buchrieser C. Specific real-time PCR for simultaneous detection and identification of *Legionella pneumophila* serogroup 1 in water and clinical samples. Appl Environ Microbiol. 2011 Mar;77(5):1708-17. doi: 10.1128/AEM.02261-10. Epub 2010 Dec 30. PMID: 21193672; PMCID: PMC3067292.

Räsänen N.H.J., Rintala H., Miettinen I.T., and Torvinen E.. 2013. Comparison of culture and qPCR methods in detection of mycobacteria from drinking waters. Canadian Journal of Microbiology. 59(4): 280-286. doi: 10.1139/cjm-2012-0695

Torvinen E, Torkko P, Rintala AN. Real-time PCR detection of environmental mycobacteria in house dust. J Microbiol Methods. 2010 Jul;82(1):78-84. doi: 10.1016/j.mimet.2010.04.007. Epub 2010 Apr 28. PMID: 20434494.
